# Roles of Uridine Diphosphoglucuronosyltransferase 2B Enzymes in Cancer Susceptibility and Treatment: A Review

**DOI:** 10.3390/ph19071016

**Published:** 2026-06-30

**Authors:** Suresh Kumar Srinivasamurthy, Vijaya Paul Samuel, Tarig Hakim Merghani Hakim, Biji Thomas George, Grisilda Vidya Bernardt, Ashwin Kamath, Chakradhara Rao Satyanarayana Uppugunduri

**Affiliations:** 1 Department of Pharmacology, RAK College of Medicine, RAK Medical and Health Sciences University, Ras Al Khaimah, United Arab Emirates; suresh@rakmhsu.ac.ae; 2 Department of Anatomy, RAK College of Medicine, RAK Medical and Health Sciences University, Ras Al Khaimah, United Arab Emirates; vijaypaul@rakmhsu.ac.ae; 3 Department of Physiology, RAK College of Medicine, RAK Medical and Health Sciences University, Ras Al Khaimah, United Arab Emirates; tarig@rakmhsu.ac.ae; 4 Department of Surgery, RAK College of Medicine, RAK Medical and Health Sciences University, Ras Al Khaimah, United Arab Emirates; biji@rakmhsu.ac.ae; 5 Department of Biochemistry, RAK College of Medicine, RAK Medical and Health Sciences University, Ras Al Khaimah, United Arab Emirates; grisilda@rakmhsu.ac.ae; 6 Department of Pharmacology, Kasturba Medical College Mangalore, Manipal Academy of Higher Education, Manipal, India; ashwin.kamath@manipal.edu; 7 Department of Medical Oncology, Jawaharlal Institute of Postgraduate Medical Education and Research, Puducherry, India

**Keywords:** cancer, *UGT2B*, *UGT2B10*, *UGT2B7*, *UGT2B17*, *UGT2B15*, cancer drugs, uridine diphosphate-glucuronosyltransferases

## Abstract

Uridine diphosphate glucuronosyltransferase 2B (*UGT2B*) enzymes constitute a critical subgroup of phase II metabolizing enzymes that modulate the clearance of steroid hormones, carcinogens, and numerous anticancer agents, thereby influencing cancer susceptibility, progression, and therapeutic outcomes. This review provides a comprehensive synthesis of the genetic, regulatory, and functional roles of *UGT2B* family members, particularly *UGT2B4*, *UGT2B7*, *UGT2B10*, *UGT2B15*, *UGT2B17*, and *UGT2B28*, in oncogenesis and cancer treatment. We summarize evidence from molecular, epidemiological, pharmacogenetic, and clinical studies demonstrating how *UGT2B* expression patterns, polymorphisms, copy number variations, epigenetic regulation, and microRNA-mediated control shape intratumoral hormone homeostasis, carcinogen detoxification, and drug resistance across multiple malignancies, including prostate, breast, lung, colorectal, hematological, and hormone-dependent cancers. *UGT2B* enzymes metabolize several widely used anticancer drugs and active metabolites, thereby affecting pharmacokinetics, efficacy, and toxicity. Understanding the context-specific roles of *UGT2B* family members offers a compelling opportunity for therapeutic exploitation. In particular, rational combination strategies incorporating *UGT2B* inhibitors or modulators alongside standard anticancer agents may enhance drug effectiveness without increasing dosage, while simultaneously enabling the dose reduction of the partner agent to mitigate dose-dependent toxicities. Such approaches are especially relevant for therapies with narrow therapeutic indices. Overall, this review highlights *UGT2B* enzymes as multifunctional determinants of cancer risk and treatment response and underscores their promise as biomarkers and actionable targets for precision oncology and optimized combination regimens.

## 1. Introduction

Uridine diphosphate-glucuronosyltransferases (UGTs) are an important family of phase II metabolic enzymes that conjugate glucuronic acid to lipophilic substrates, thereby facilitating their enhanced solubility and elimination [1]. These enzymes play a vital role in the biotransformation of endogenous substances and xenobiotics, underscoring their broad physiological significance [1,2]. Within the UGT superfamily, the *UGT2B* subfamily is particularly relevant in oncology due to its involvement in the metabolism of steroid hormones, bile acids, and xenobiotics [2,3]. Six isoforms have been identified in this subfamily: *UGT2B4*, *UGT2B7*, *UGT2B10*, *UGT2B11*, *UGT2B15*, and *UGT2B17* [2,3]. Except for *UGT2B10* and *UGT2B11*, all isoforms have demonstrated activity toward steroid molecules [4].

*UGT2B7* most efficiently glucuronidates estrogens, catechol estrogens, and androstane3α,17βdiol [4]. *UGT2B15* and *UGT2B17* display similar activity toward androstane3α,17βdiol (approximately 30% lower than *UGT2B7*), while *UGT2B17* shows the highest activity toward androsterone, testosterone, and dihydrotestosterone. *UGT2B4* also acts on 5α-reduced androgens and catechol estrogens, though with lower efficiency [4].

A comprehensive analysis of UGT expression across 23 human tissue types revealed several UGT isoforms in steroidogenic tissues such as the breast, prostate, heart, and adrenal glands [5]. However, UGT enzymes are predominantly expressed in the alimentary tract and liver [5]. Notably, *UGT2B4* and *UGT2B15* exhibit exceptionally high hepatic expression, with levels approximately nine-fold and four-fold higher, respectively, than *UGT2B7* [5].

In the prostate, *UGT2B* enzymes localize mainly to epithelial cells, strongly in basal and moderately in luminal cells, with *UGT2B17* found exclusively in basal cells [6]. *UGT2B17* converts DHEA to androsterone and 3αdiol, while *UGT2B15*, localized in luminal cells, acts where DHT is formed from testosterone [7]. This spatial expression pattern underscores their role in inactivating potent androgens and protecting prostate tissue from excessive steroid activity [6,7].

The selective expression of *UGT2B* enzymes suggests organ-specific roles, with presence not only in the liver but also in extrahepatic tissues such as the prostate, breast, and ovary, where they regulate local steroid levels [8,9]. *UGT2B15* and *UGT2B17* are key enzymes that conjugate 5α-reduced C19 steroids, including dihydrotestosterone (DHT), in prostate cells. Their expression is differentially regulated by hormones and growth factors, with *UGT2B17* being more labile than *UGT2B15* [8]. Overexpression of *UGT2B17* in prostate cells reduces androgen responsiveness by enhancing steroid clearance [8]. Protein stability studies revealed that *UGT2B17* is the most labile enzyme [4]. *UGT2B17* is also the first human UGT enzyme demonstrated to be found in extrahepatic tissues, such as the prostate and uterus [8].

Genetic variation in UGT enzymes affects the in vivo glucuronidation of tobacco-related compounds, a key process in detoxifying nicotine and carcinogenic nitrosamines [10]. The strongest associations were found with *UGT2B10* variants, particularly for cotinine glucuronidation in two ethnic groups studied (e.g., rs2331559, rs11726322 in Europeans; rs835309 in African Americans) [10]. Other nominal associations involved UGT2B17*2, *UGT2B7*, and *UGT1A* variants with the glucuronidation of nicotine, trans-3′-hydroxycotinine (3HC), and 4-(methylnitrosamino)-1-(3-pyridyl)-1-butanol (NNAL). Thus, *UGT2B10* plays a pivotal role in cotinine metabolism, and genetic differences in UGT enzymes contribute to ethnic variability in glucuronidation capacity, potentially explaining smoking-related cancer risk disparities [10].

Beyond their metabolic roles, evidence increasingly supports the involvement of *UGT2B* enzymes in cancer-related processes [3]. Differential UGT expression has been associated with the initiation and progression of oncogenic signaling pathways, contributing to tumor development [3,9,11]. The large-scale cancer genome atlas (TCGA) analysis of 10,069 tumors across 33 cancer types identified 3427 somatic mutations in *UGT* genes, with about 18% of tumors harboring such mutations [12]. Approximately 65% of these mutations were coding (missense, nonsense, or frameshift), while 10% occurred in noncoding regions, potentially impacting gene regulation [12]. Mutation frequency varied widely across cancers, exceeding 25% in colon, lung, skin, and uterine cancers, but under 5% in others. The frequently affected gene is *UGT2B4*.

Recent molecular profiling and bioinformatic analyses have identified *UGT2B15* as an oncogene, with overexpression linked to poor patient outcomes in gastric cancer, potentially mediated through the HippoYAP signaling pathway [13]. The *UGT2B* genes exhibit numerous single-nucleotide polymorphisms and cis-regulatory variants [14]. Notably, *UGT2B17* and *UGT2B28* exhibit common whole-gene deletion polymorphisms [15].

In the context of cancer chemotherapy, UGTs are involved in the metabolism of anticancer agents or their metabolites, such as topoisomerase inhibitors, tyrosine kinase inhibitors, and hormonal modulators [16,17,18]. The cellular UGT activity also influences resistance to anticancer agents [19,20]. Dellinger et al. showed that UGT expression diminishes during melanoma progression [19]. However, metastatic melanoma cell lines treated with vemurafenib showed the re-expression of *UGT2B7*, *UGT2B10*, and *UGT2B15*, accompanied by a corresponding increase in glucuronidation activity—indicating an adaptive metabolic response to therapy [19]. Silencing *UGT2B7* increased sensitivity to adriamycin and epirubicin, suggesting a role in drug resistance [19]. Additionally, cancer cells such as CLL may express high *UGT2B17*, locally inactivating fludarabine and affecting intratumoral drug levels [20]. The current review presents a narrative synthesis of the role of *UGT2B* in cancer, focusing on genetic or functional variation, cancer type, and therapeutic implications.

A comprehensive literature search was performed using MEDLINE, Scopus, and Web of Science databases to identify relevant English language studies published from database inception to early 2026. Search terms combined keywords such as “Phase II glucuronosyl conjugation enzymes,” “*UGT2B* family enzymes,” “UDP-glucuronosyltransferases 2B family,” “cancer predisposition,” and “oncology treatment,” connected with Boolean operators (AND, OR). Studies were included if they addressed the role of *UGT2Bs* in cancer susceptibility, association, progression, or therapeutic response, encompassing both in vitro and clinical evidence. Exclusion criteria included non-English publications, conference abstracts without full text, and studies lacking mechanistic or pharmacological relevance. Studies were selected based on title and abstract screening, followed by full-text review. Data were extracted and summarized narratively to identify key findings, mechanisms, and gaps in the literature. Given the narrative design, the focus was on the conceptual synthesis of key evidence and mechanisms.

## 2. *UGT2B* Structural Characteristics

The *UGT2B* gene cluster on chromosome 4q13 was mapped, containing *UGT2B4, UGT2B7, UGT2B15*, pseudogenes, and remnant gene fragments, with *UGT2B4* located between *UGT2B7* and *UGT2B15* [21]. Also, the *UGT2B* gene family is hypothesized to have evolved through recent gene duplication, mutation, and rearrangement events, showing greater genetic complexity and diversity [21]. Protein sequence similarity among the *UGT2B* family members is moderate to high, with few members sharing more than 90% similarity. Despite sharing 95% sequence identity, the *UGT2B15* and *UGT2B17* genes showed differences in basal promoter activity, indicating distinct regulatory mechanisms [22].

### 2.1. UGT2B Substrates with Oncological Relevance

*UGT2B* members plays a critical role in phase II detoxification of carcinogens like NNAL and steroid compounds (Table 1). The Bisphenol A (BPA), a known endocrine disruptor, is primarily glucuronidated through *UGT2B15* in human liver microsomes [23]. Comparative kinetic and inhibition studies showed substrate-selective glucuronidation by different *UGT2B* enzymes. *UGT2B7* selectively glucuronidated 6α-hydroxyprogesterone and 21-hydroxyprogesterone, with 6α-OHP following Michaelis–Menten kinetics and 21-OHP showing positive cooperativity [24]. *UGT2B7* also mediated the high-affinity glucuronidation of 11α-hydroxyprogesterone. In contrast, *UGT2B15* and *UGT2B17* were the primary enzymes responsible for testosterone 17β-glucuronidation and the high-affinity glucuronidation of 16α-hydroxyprogesterone. Molecular docking and molecular dynamics simulations identified acetaminophen, lorazepam, mycophenolic acid, and a voriconazole N-oxide intermediate as potential ligands of *UGT2B10*, which may have significance for drug interactions [25].

A pharmacophore model identified key structural features required for intestinal *UGT2B17* substrates, including an accessible hydroxyl or carboxyl group, a nearby hydrophobic group, and an aromatic ring [26]. However, several more tested compounds, including amines, inhibited *UGT2B17* regardless of whether they were substrates, suggesting multiple inhibitory mechanisms. Thus, *UGT2B17* has broad substrate and inhibitor promiscuity, contributing to variability in oral drug metabolism and disposition [26]. Precht et al. identified the carbinol metabolite of the phase 1 reaction of letrozole metabolism as a novel in vitro probe substrate for *UGT2B7* [27].

**Table 1:** *UGT2B* members and their substrates, modulators in the context of oncology.

UGT Isoform(s)	Substrates	Inducers	Inhibitors	Oncology Relevance	References
*UGT2B4*	Hyodeoxycholic acid (HDCA), catechol estrogens, codeine, morphine, canagliflozin, carvedilol, clopidogrel carboxylate, propranolol	Fenofibric acid, Chenodeoxycholic acid	Clotrimazole, methadone	Important in bile acid and endogenous steroid detoxification	[28,29,30,31,32,33,34,35]
*UGT2B7*	Androsterone, catechol estrogens, estriol, steroids, fatty acids, 4 OH tamoxifen, endoxifen, epirubicin, letrozole, hydroxycotinine, HDCA, 6α-hydroxyprogesterone, 21-hydroxyprogesterone, 11α-hydroxyprogesterone, zidovudine, morphine, hydromorphone, codeine, buprenorphine, ketoprofen, all-trans retinoic acid, efavirenz, R-oxazepam, carvedilol, clopidogrel carboxylate, propranolol, lamotrigine, chloramphenicol, asciminib	Dexamethasone, rifampin	Tetrahydrocannabinol (THC) and cannabidiol (CBD), mefenamic acid, licoagrochalcone A, glycycoumarin, crizotinib, ceritinib, asciminib, everolimus, bromophenols, fluconazole, flavone O-glycosides, methadone	Metabolism of endogenous steroids and bile acids Pharmacokinetics of letrozole and tamoxifen Drug interactions of oncology context—crizotinib, ceritinib, asciminib, everolimus, cannabidiol and morphine	[24,27,28,29,30,34,36,37,38,39,40,41,42,43,44,45,46,47,48,49,50,51,52,53,54,55,56,57,58,59]
*UGT2B10*	Cotinine, nicotine, NNAL, amitriptyline, imipramine, clomipramine, trimipramine, medetomidine, clozapine, olanzapine, dexmedetomidine	Aflatoxin B1	lorazepam, mycophenolic acid, fluconazole, amitriptyline, doxepin, mianserin, desloratadine, loratadine	N-glucuronidation; polymorphisms affect nicotine dependence and smoking behavior	[60,61,62,63,64,65,66,67]
*UGT2B15*	Sorafenib, dasatinib, imatinib, 4 OH tamoxifen, DHT, androsterone, 5αandrostane 3α, 17βdiol, androsterone, 16α-hydroxyprogesterone, S-oxazepam, lorazepam, rofecoxib, acetaminophen, hydroxyphenytoin (R isomer), morphine, dabigatran, bisphenol A	Naftopidil, Aflatoxin B1	CBD, 11-OH-THC, and THC, entrectinib, tucatinib, fedratinib, pexidartinib, calcitriol	Prostate androgen clearance; variants linked to prostate cancer	[23,24,36,38,46,67,68,69,70,71,72,73,74,75,76,77,78]
*UGT2B17*	Testosterone, DHT, androsterone, 5αandrostane 3α,17βdiol, 16α-hydroxyprogesterone, 3hydroxycotinine, exemestane, fludarabine, vorinostat, morphine, clopidogrel carboxylate, diclofenac, asciminib	Exemestane, 17-hydroyexemestane, Aflatoxin B1	Imatinib, tucatinib, asciminib, curcumin, salicylic acid, calcitriol	Glucuronidation of 17dihydroexemestane High *UGT2B17* in leukemic cells inactivates fludarabine, contributing to drug resistance UGT2B17*2 genotype is associated with reduced glucuronidation of vorinostat	[24,29,51,67,75,78,79,80,81,82,83,84,85,86,87]

*UGT2B4* is highly liable for differential expression through alternative promoters, exon skipping, and alternative splicing [11]. Sorafenib, dasatinib, and imatinib showed mixed inhibition of paracetamol glucuronidation, while demonstrating strong inhibitory effects on *UGT1A9* and *UGT2B15* [69].

Sun et al. demonstrated that multiple UGTs, particularly *UGT2B7* in addition to *UGT1A10* and UGT1A8, are significantly involved in 4 OH tamoxifen and endoxifen metabolism [36]. Furthermore, fluconazole, a recognized *UGT2B7* inhibitor, markedly reduced efavirenz (EFV) glucuronide formation by up to 80%, indicating that EFV may serve as a specific *UGT2B7* substrate in vitro [37]. Flunitrazepam (FNZ) inhibited *UGT2B7*-mediated catechol estrogen glucuronidation and also inhibited buprenorphine glucuronidation by *UGT1A1* and *UGT2B7* but not *UGT1A3* [39].

Similarly, cotinine was identified as a highly selective substrate for *UGT2B10*-mediated N-glucuronidation [66]. Among phenotyping inhibitors, only fluconazole significantly inhibited *UGT2B10* activity. Most antidepressants and antipsychotics tested inhibited *UGT2B10*, with the strongest inhibition observed for amitriptyline, doxepin, mianserin, desloratadine, and loratadine [66]. In vitro in vivo prediction showed clinically significant interactions with medications exclusively metabolized by *UGT2B10*.

Innocenti et al. (2001) studied the specific UGT isoform responsible for epirubicin glucuronidation in humans [43]. The methodology utilized the screening of seven UGT isoforms (*UGT1A1*, 2B7, 2B15, etc.) in cellular systems and the analysis of microsomes from 47 human livers and HK293 cells expressing *UGT2B7* variants. It was concluded that *UGT2B7* is the major human UGT catalyzing epirubicin glucuronidation, evidenced by its unique activity among screened isoforms, having an oncological relevance [43].

### 2.2. The UGT2B Inhibitors and Inducers

The *UGT2B* family of glucuronosyltransferases is widely modulated by xenobiotics that are not themselves substrates. For example, zafirlukast exhibits substrate-specific inhibition of UGTs, particularly UGT1As, and a moderate inhibitor of *UGT2Bs* [88]. Exposure to peroxisome proliferator-activated receptor (PPAR) α activators (fenofibric acid) has been shown to significantly elevate *UGT2B4* mRNA expression in hepatocytes or hepatoblastoma HepG2 and Huh7 cells, thereby potentially increasing the catabolism of bile acids [31].

PPARα agonists such as fenofibric acid promote cytotoxic bile acid catabolism apart from their role in lipid and cholesterol metabolism in human hepatocytes, through the induction of *UGT2B4* [31]. Chenodeoxycholic acid, apart from being a substrate, also independently induces *UGT2B4* through its agonism on farnesoid X receptor (FXR) [89]. By developing a selective activity assay using canagliflozin, Lapham et al. identified clotrimazole as a potent and highly selective inhibitor of *UGT2B4* [32]. The metabolic activity of *UGT2B7* was enhanced in HuH-7 cells by dexamethasone, possibly via glucocorticoid receptor activation [48]. Exemestane upregulated *UGT2B17* expression in breast cancer cells through steroid-responsive promoter elements. Licoagrochalcone A and Glycycoumarin were also identified as potent *UGT2B7* inhibitors using UPro2B7-enabled real-time imaging of endogenous *UGT2B7* activity [49]. Among Anaplastic lymphoma kinase (ALK) inhibitors, used for ALK-positive non-small cell lung cancer (NSCLC), crizotinib and ceritinib have shown inhibitory activity to *UGT2B7* [50]. Asciminib, used for adult patients with chronic-phase chronic myeloid leukemia harboring the T315I mutation, is also a substrate of *UGT2B7* and *UGT2B17* [51]. In vitro enzyme-based assays using human liver microsomes demonstrated that asciminib inhibited *UGT2B7* [51]. Everolimus, an inhibitor of mammalian target of rapamycin (mTOR), is also shown to exhibit moderate inhibitory activity to *UGT2B7* [52]. Another example is that of cannabinoids inhibiting *UGT2B7*-mediated metabolism of opioids, indicating a clinically relevant risk of moderate drug–drug interactions when opioids are used alongside cannabis- or cannabinoid-based therapies [45]. Salicylic acid inhibited *UGT2B17* activity through an uncompetitive inhibition mechanism, which has negligible potential to cause clinically significant drug interactions [86].

Environmental pollutants, such as Bromophenols (BPs), were also demonstrated to potently inhibit several UGT enzymes, including *UGT2B7*, using glucuronidation assays [53]. Using recombinant UGT-mediated 4-methylumbelliferone glucuronidation assays, Yang et al. demonstrated that both Polycyclic aromatic hydrocarbons (PAHs) and hydroxylated metabolites (OH-PAHs) inhibited several UGT isoforms, including UGT1A6, *UGT1A9*, and *UGT2B7* [90]. Similarly, HPLC-based 4-methylumbelliferone disposition assays demonstrated that dietary flavone O-glycosides such as rutin and nicotifiorin significantly inhibited recombinant *UGT2B7* activity [55]. Similarly, the enantiomers of naftopidil (NAF), an α_1_D/α_1_A adrenoceptor antagonist, upregulate *UGT2B15* in human BPH1 cells [91]. This is accompanied by reduced intraprostatic and intracellular DHT levels and consequent increase in apoptosis. These findings suggest that NAF enantiomers act as novel *UGT2B15* inducers, distinct from AR antagonists and 5α-reductase inhibitors, by enhancing DHT elimination and promoting apoptosis in prostate cells [91].

Several tyrosine kinase inhibitors (TKIs) exhibited inhibitory potential towards several *UGT2B* family members. Entrectinib used in non-small cell lung cancer (NSCLC) has shown inhibition of *UGT2B15* in recombinant in vitro assays [74]. Fedratinib, approved for myelofibrosis, potently inhibited *UGT2B15* in enzyme kinetics systems and human liver microsomes (HLMs) [76]. Pexidartinib approved for adult tenosynovial giant cell tumor was shown to act as a broad inhibitor of multiple human UGT enzymes in recombinant in vitro assays [77]. It showed mixed inhibition of *UGT2B15*, while in vitro–in vivo extrapolation (IVIVE) analysis predicted a significant risk of drug–drug interactions with UGT-metabolized drugs at clinical doses [77]. Imatinib was also demonstrated to inhibit *UGT2B17* in human enzyme assays [84]. Tucatinib, approved for adult advanced unresectable or metastatic HER2-positive breast cancer, showed in vitro inhibition of multiple human UGT enzymes, including *UGT2B15* and *UGT2B17*, suggesting potential clinically relevant drug–drug interaction risk and the possibility of exploitation of this drug interaction for therapeutic efficacy [75].

Natural products may also inhibit *UGT2B* isoforms. Bacchav et al. demonstrated that curcumin inhibited *UGT2B17*-mediated testosterone glucuronidation in human intestinal cells, significantly reducing testosterone glucuronide and androstenedione formation [85]. The in vitro findings were supported by a pilot crossover study in hypogonadal men, which showed that the coadministration of curcumin with oral testosterone undecanoate increased testosterone bioavailability [85]. Treatment with Aflatoxin B1 significantly upregulated the mRNA expression of *UGT1A3*, *UGT2B10*, *UGT2B15*, and *UGT2B17* in HepG2 cells [67].

Endogenous molecules were also shown to modulate the activity of *UGT2B* isoforms in addition to being a substrate of these enzymes. β-Estradiol (β-E2) and α-estradiol (α-E2) differ in the stereochemical configuration of the C17-OH group, which markedly influences their interactions with UGT enzymes. β-E2 moderately inhibited *UGT1A9*, *UGT2B4*, and *UGT2B7*, while enhancing *UGT2B17* activity by improving substrate binding and catalytic turnover. In contrast, α-E2 showed much stronger inhibition of *UGT2B4* and *UGT2B7*, but had minimal effects on *UGT1A9* and *UGT2B17* [92]. Calcitriol, a vitamin D receptor activator, negatively regulates *UGT2B15* and *UGT2B17* in prostate cancer LNCaP cells, reducing androgen inactivation.

## 3. Genetic Factors Influencing the *UGT2B* Genes, Expression and Function

UDP-glucuronosyltransferases (UGTs) are regulated at multiple biological levels. At the genetic level, polymorphisms such as single-nucleotide polymorphisms (SNPs) and copy number variations (CNVs) can affect gene structure and expression [93]. Transcriptionally, promoter activity and transcription factor binding influence the initiation and rate of UGT gene transcription. At the post-transcriptional level, UGT regulation includes alternative splicing, which generates diverse isoforms with potentially distinct functions, and microRNAs (e.g., miR376), which bind to UGT mRNAs to modulate their stability and translation. Translational regulation involves ribosomal control and RNA binding proteins that influence how efficiently UGT mRNAs are translated into proteins. Finally, posttranslational modifications affect UGT protein stability, subcellular localization, and interactions, thereby fine-tuning enzyme activity and function. The list of common genetic variants reported in *UGT2B* family members and their minor allele frequencies across different populations is outlined in Supplementary Material Table S1. These population differences indicate their relevance in defining the therapeutic outcomes of substrates of the *UGT2B* family. These differences are also essential to understand the exploitation of drug–drug interactions and their relevance in specific populations.

Neumann et al. showed that the expression of UGTs, including *UGT2B7* and *UGT2B17*, increased throughout childhood and adolescence, potentially influenced by hormonal signaling [94]. This developmental regulation may contribute to interindividual variability in drug response among pediatric patients [94]. The elevated UGT activity including that of *UGT2B7* seemed to explain a significant portion of the metabolic changes observed during pregnancy which has impact for analgesics use such as buprenorphine [95]. Studies have investigated genetic factors influencing testosterone/epitestosterone (T/E) ratios, a key marker in doping tests. While a T/E ratio > 4 suggests testosterone abuse, some individuals naturally exceed this threshold, risking false positive results [96]. T/E ratio analyses in urine samples and the genotyping of men for polymorphisms in *CYP17, UGT2B17, UGT2B15*, and *UGT2B7* showed that individuals lacking *UGT2B17* had higher *UGT2B15* mRNA expression, suggesting a compensatory mechanism [96]. These findings highlight that genetic variation in *UGT2B17*, at least partly, can affect T/E ratios.

**Figure 1:**
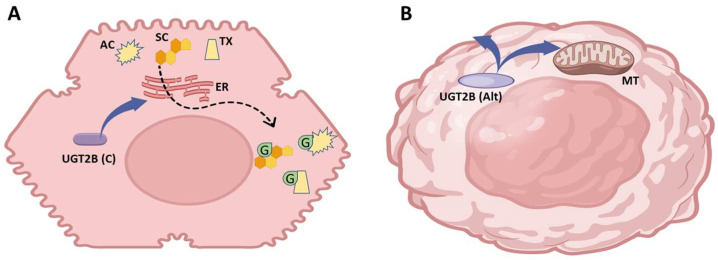
Contrasting functional roles of *UGT2B* canonical and alternative isoforms in hepatocytes (**A**) and cancer cell (**B**). *UGT2B* (C) represents canonical uridine 5′-diphospho-glucuronosyltransferase 2B isoforms localized to the endoplasmic reticulum (ER), responsible for the glucuronidation (G) of anticancer drugs (AC) and steroid compounds (SC) and toxicants (TX) in hepatocytes. *UGT2B* (Alt) indicates alternative splice isoforms, which may have reduced or no enzymatic activity, but are involved in cellular adhesion and metabolic pathways in mitochondria (MT) of cancer cells.

Lampe et al. examined the frequency of *UGT2B4*(D458E), *UGT2B7*(H268Y), and *UGT2B15*(D85Y) polymorphisms in 233 individuals of Asian and Caucasian descent [97]. Significant ethnic differences were found in allele and genotype frequencies for all three polymorphisms (*p* < 0.02). Asians showed a much higher frequency of *UGT2B4* (D458) (100%) and a greater occurrence of *UGT2B7*(H268) and *UGT2B15*(D85) homozygosity compared to Caucasians [97]. Conversely, Caucasians had a higher prevalence of Y268 and Y85 homozygotes. These findings suggest notable interethnic variability in *UGT2B* gene polymorphisms, which probably contributes to differences in hormone metabolism and cancer susceptibility, particularly in sex hormone-related cancers, highlighting the need for further multiethnic research (see Supplementary Table S1). Nuclear receptors, such as pregnane X receptor (PXR), constitutive androstane receptor (CAR), and PPAR, play a role in the regulation of UGTs, which is specific to tissues [98]. Alternative splice variants, often catalytically inactive, can regulate enzyme activity and are implicated in tumor progression in cancer cells [99] (Figure 1).

## 4. UGT 2B Family and Their Role in Various Cancers

### 4.1. UGT 2B and Prostate Cancer

Prostate cancer is the most studied in association studies evaluating *UGT2B* polymorphisms risks (Table 2). Androgens play a pivotal role in prostate physiology and cancer progression. Active androgens like dihydrotestosterone (DHT) are metabolized and cleared through glucuronidation by *UGT2B15* and *UGT2B17*. Several studies have shown positive association between *UGT2B* and prostate cancer [68,100,101,102,103,104,105,106,107,108,109]. Park et al. in a case–control (420 prostate cancer patients and 487 controls) genetic study involving African American and Caucasian showed that *UGT2B17* null genotype (gene deletion) was linked to an augmented risk of prostate cancer, especially in Caucasians, but not significantly in African Americans [104]. Studies have also explored the additive effects of UGT with other polymorphisms. Age-matched case–control study from the United States (356 prostate cancer patients and 363 controls) demonstrated that the *UGT2B17* null polymorphism was significantly associated with increased risk (OR = 1.7), and the risk was even higher in individuals with the HSD3B1 Asn/Asn genotype (OR = 2.7) [106]. Similarly, a study investigated the biochemical recurrence (BCR) of prostate cancer after radical prostatectomy (RP) in relation with UGT gene polymorphisms, particularly *UGT2B17* and *UGT2B28* deletions [107]. Among 526 Caucasian and 320 Asian men with localized prostate cancer, those with at least two deleted *UGT2B* gene copies had a significantly higher risk of BCR (HR, 2.26 and 2.16, respectively) [107]. In Asians, *UGT2B17* deletion was also linked to higher Gleason scores, while no associations were found with PSA or TNM staging. Patients with these deletions also showed reduced circulating androgen glucuronides, indicating altered androgen metabolism. This study suggested a potential prognostic value of inherited UGT deletions in prostate cancer progression, indicating that such genetic variants may influence recurrence risk through their impact on hormonal inactivation pathways [107].

However, not all studies have shown an unequivocal positive result [110,111,112,113,114,115]. A large population-based case–control study found no association between the *UGT2B17* gene deletion polymorphism and prostate cancer risk or prostate cancer-specific mortality, contradicting earlier reports [111]. Similarly, a study evaluated *UGT2B17* gene deletion polymorphism in a Caucasian population [113]. Using a high-throughput real-time PCR genotyping method, researchers analyzed 411 prostate cancer cases and 397 controls. The frequency of the *UGT2B17* homozygous deletion (0/0) was 12% among controls, aligning with expected population frequencies. However, there was lack of significant association between the deletion polymorphism and prostate cancer risk [113]. Hu et al. identified a functional promoter polymorphism (155G/A) in the *UGT2B17* gene, which influences its expression in prostate cells. FOXA1 knockdown reduced *UGT2B17* expression, and individuals with the 155A allele had higher levels of glucuronidated androgen metabolites in circulation. Despite its functional effect on gene regulation, the 155G/A polymorphism was not associated with prostate cancer risk [114]. Also, another study found no significant association between the analyzed polymorphisms and prostate cancer (PC) risk in Iranian patients; however, it showed increased risk for benign prostatic hyperplasia (BPH) [115].

A meta-analysis of six studies by Kpoghomou et al., including 7029 participants (3839 prostate cancer cases and 3190 controls), evaluated the relationship between *UGT2B17* gene status and PC risk [116]. The pooled analysis revealed a significant association, with individuals carrying the *UGT2B17* deletion showing an increased risk of PC (OR = 1.74, 95% CI: 1.14–2.64, *p* < 0.001). Subgroup analyses by ethnicity and control type yielded consistent results, confirming that *UGT2B17* genetic variation contributes to prostate cancer susceptibility [116]. A comprehensive meta-analysis evaluating the association of *UGT2B17* deletion and *CYP17* T34C polymorphisms with PC risk analyzed data from over 25 studies, involving approximately 17,000 subjects [117]. The analysis found a lack of significant association between the *CYP17* c34T>C polymorphism and prostate cancer across various genetic models. However, the *UGT2B17* deletion polymorphism (Del/Del genotype) showed a marginal association with increased prostate cancer risk in the overall analysis, and a statistically significant association. These findings reinforce the functional importance of *UGT2B17* in androgen metabolism and suggest a potential genetic susceptibility marker for PC [117].

### 4.2. UGT 2B and Breast Cancer

**Figure 2:**
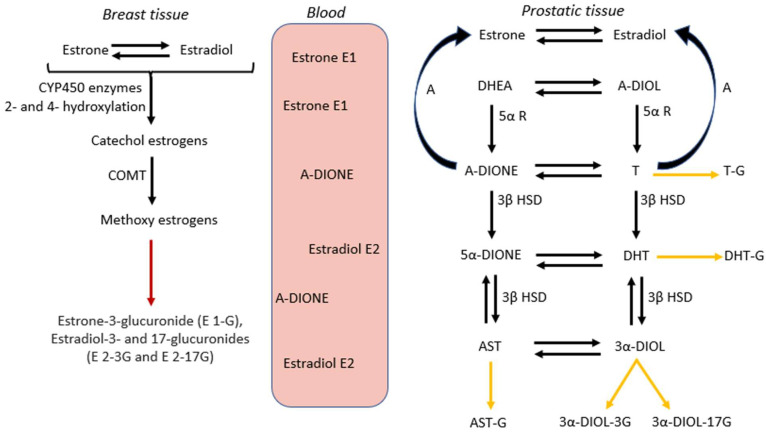
Schematic representation of steroid hormone metabolism in breast and prostate tissues, highlighting the role of *UGT2B* enzymes in androgen and estrogen glucuronidation and elimination. Red arrows mediated by *UGT1A1*, *UGT1A3*, *UGT1A10*, *UGT2B7*, *UGT2B15*, *UGT2B17*, *UGT2B28*. Yellow arrows are mediated by *UGT2B15*, *UGT2B17*. Horizontal reversible arrow symbol mediated by 17β HSD. A, Aromatase (CYP19A1); COMT, Catechol-O-Methyltransferase; A-DIONE, Androstenedione; A-DIOL, Androstenediol (usually 5-androstenediol); DHEA, Dehydroepiandrosterone; 3β-HSD, 3β-Hydroxysteroid Dehydrogenase; 5α-R, 5α-Reductase (5α-hydroxysteroid reductase); T, Testosterone; DHT, Dihydrotestosterone; 3α-DIOL, 3α-Androstanediol; AST-G, Androsterone Glucuronide.

Genetic variation upstream of the *UGT2B4* gene, a key enzyme in the metabolism of steroid hormones and bile acids, has been a focus in relation to breast cancer risk (Figure 2) [118]. A study investigated the association between tag SNP rs13129471 and breast cancer risk in 1261 patients and 825 controls of African ancestry [118]. The homozygous variant genotype was significantly associated with increased breast cancer risk, while heterozygotes showed no significant effect [118]. Similarly, a retrospective study assessed the link between *UGT2B7* gene polymorphisms and breast cancer risk in Han Chinese women [119]. Among 672 breast cancer patients and 670 healthy controls, the rs7441774 G allele was significantly more frequent in cases (41.2% vs. 35.8%; *p* = 0.006), and individuals with the GG genotype had a notably higher risk compared to those with AA genotype (adjusted OR = 1.63, *p* = 0.008). Functional assays showed that the rs7441774 G allele reduces *UGT2B7* transcriptional activity, suggesting a potential mechanism [119]. These findings support a role for *UGT2B7* variants, particularly rs7441774, in breast cancer susceptibility in the Chinese population. Contrary to these reports, no association of *UGT2B7* polymorphisms with breast cancer was reported in Jordanian Arab women [120].

*UGT2B* genetic variants increases the risk for breast cancer by altering circulating sex hormone levels [121]. Among 163 women in western Washington, the *UGT1A1* variant allele was significantly associated with a reduced risk of estrogen receptor-negative tumors, while *UGT2B15* and *SULT1A1* variants showed non-significant protective trends [121]. In a subset of postmenopausal patients not on hormone therapy, estradiol levels were significantly higher in those with *UGT2B15* Asp/Tyr or Tyr/Tyr genotypes [121]. Functional polymorphisms in *UGT2B* were not associated with sex hormone clearance, and with breast density, in premenopausal women aged 40–45 [122].

In addition to genetic variants gene expression, changes may alter the risk for breast cancer. Haakensen et al. investigated the biological basis of mammographic density (MD) for breast cancer through the gene expression profiling of breast tissue from 143 women (79 healthy and 64 with breast cancer) [123]. Analysis revealed that 24 genes, including three UGT genes and ESR1 (estrogen receptor), were downregulated in high MD tissues [123]. Notably, *UGT2B10* expression independently predicted MD, regardless of age, hormone therapy, or parity. These findings suggest that lower UGT expression is associated with higher MD and may contribute to increased breast cancer risk, particularly in younger women and hormone therapy users [123]. Another case–control study (236 breast cancer patients and 203 controls) in an Iranian population demonstrated significant association of *UGT2B17* null genotype with breast cancer [124].

### 4.3. UGT2B and Lung Cancer

*UGT2B17*, known to metabolize tobacco-related carcinogens like NNAL, exhibits common copy number variation CNVs [125]. However, the null genotype was not significantly more frequent among lung cancer patients, including women with adenocarcinoma, and showed no association with overall survival or cancer risk by sex or tumor type. Thus, there is a need for the population-specific validation of genetic risk markers [125].

Qian et al. showed that the rs12233179 SNP of *UGT2B7* was associated with an increased risk of non-small cell lung cancer (NSCLC) in never-smoking Chinese women [126]. Furthermore, *UGT2B7* was found to be upregulated in the tumor tissues of female NSCLC patients and was linked to a poor prognosis [126]. Given that the SNP is involved in the altered regulation of sex hormone binding globulin, a potential involvement of sex steroid hormones in lung cancer has been proposed.

### 4.4. UGT2B and Esophageal Cancers

Hu et al. investigated the contribution of CNVs to esophageal squamous cell carcinoma (ESCC) risk using a candidate gene strategy [15]. Among six CNVs examined in 404 ESCC patients and 402 controls, a significant association was observed between increased ESCC risk and *UGT2B28* copy number loss. This germline copy number loss was also associated with somatic gene deletions and reduced *UGT2B28* mRNA levels in tissues, indicating impaired detoxification capacity. These findings substantiate the role of *UGT2B* in the detoxification of carcinogens such as NNAL [127]. Furthermore, it should be noted that steroid compounds, including bile acids and sex steroids, are known risk factors for ESCC [128,129].

Similarly, a study by Lian et al., exploring the common pathways between ESCC and esophageal adenocarcinoma (EAC), found that *UGT2B17* and miR224, originally linked to ESCC, was also significantly associated with EAC. Functional analysis suggested shared biological pathways between the two cancer types. Notably, increased *UGT2B17* expression and reduced miR224 signaling may influence EAC development and its responsiveness to male sex hormones [130].

The case–control study by Dura et al., among 351 EC patients and 592 controls, showed that *UGT1A1* high-activity genotypes were linked to risk of ESCC (OR = 1.62), possibly due to lower bilirubin (an antioxidant) [131]. In contrast, *UGT1A8* and *UGT2B4* genotypes linked to reduced predicted enzyme activity were significantly associated with a higher risk of ESCC, likely due to the impaired detoxification of toxic carcinogens [131]. No associations were found for EAC. These findings also highlight the role of the *UGT2B*-related detoxification capacity of carcinogens in ESCC susceptibility.

Dura et al. studied 351 esophageal cancer patients and 592 controls to determine the functional polymorphisms in *UGT* genes, which influence detoxification enzyme activity, affecting EC risk [131]. Based on predicted in vivo enzyme activity, UGT genotypes were classified as high, medium, or low activity. Significant associations were found for ESCC but not EAC. Specifically, high-activity *UGT1A1* genotypes were linked to increased ESCC risk (OR = 1.62), whereas high-and-medium-activity *UGT2B4* and high-activity *UGT1A8* genotypes were associated with reduced ESCC risk [131]. The findings suggest that UGT polymorphisms may modulate susceptibility to ESCC, likely through effects on carcinogen detoxification and antioxidant metabolism, but do not influence EAC risk.

### 4.5. UGT2B and Bladder Cancer

Lin et al. investigated whether the *UGT2B7* c.802C>T (His268Tyr) polymorphism is associated with bladder cancer risk in benzidine-exposed Chinese workers from the dye industry [132]. Among 36 bladder cancer cases and control groups (251 exposed and 218 unexposed individuals), the TT genotype was significantly more frequent in cancer patients (25%) than in unexposed controls (9%; OR = 3.30, 95% CI 1.37–7.98, *p* = 0.006). The T allele was also more common in cases (46%) than in controls (33%; OR = 1.73, 95% CI 1.05–2.87, *p* = 0.03). Population comparison showed that the TT genotype was less frequent in Chinese than in Caucasians but similar to Japanese frequencies. Overall, the findings suggest that the *UGT2B7* TT genotype may increase bladder cancer susceptibility in individuals exposed to benzidine, likely due to altered detoxification capacity [132]. But this observation was not consistent across other populations [133]. Using a PCRRFLP assay, researchers analyzed 211 bladder cancer cases, 210 urological controls, and 171 occupationally exposed patients from Germany. The TT genotype frequencies were similar across all groups (27% in cancer cases, 35% in controls, 25% in exposed patients), indicating no significant association between *UGT2B7* genotype and bladder cancer risk in this Caucasian population. However, the study confirmed ethnic differences in *UGT2B7* genotype frequencies between Caucasians and Chinese, suggesting that genetic background may influence susceptibility in different populations [133].

### 4.6. UGT2B and Colorectal Cancers

The studies investigating the effect of *UGT2B17* and *UGT2B28* gene deletions on colorectal cancer (CRC) risk are sparse. In one such study in a Caucasian population, involving 665 CRC cases and 621 controls, the *UGT2B17* deletion genotype (0/0) showed a significant association with a reduced risk of CRC, particularly in men, but not in women [134]. The protective effect was especially evident for rectal cancer, with no association found for colon cancer or for *UGT2B28* CNV [134]. These findings suggest that *UGT2B17* deletion may lower CRC risk, possibly by increasing exposure to protective compounds like flavonoids and NSAIDs, which are normally metabolized by *UGT2B17*. In the same direction, a meta-analysis confirmed a significant association between the *UGT2B7* rs7439366 variant and colorectal cancer risk, suggesting that it could serve as a promising biomarker [135]. Similarly, in a study of 79 CRC cases, copy number aberration data, gene expression profiles, and clinical information highlighted several potential CRC-related genes, including *UGT2B28* [136].

Van der Logt et al. investigated whether the *UGT2B7* H268Y polymorphism influences CRC risk in 411 Caucasian CRC patients and 600 controls [137]. While overall genotype frequencies did not differ between groups, the *UGT2B7* genotype was significantly more frequent in patients with proximal colon cancer (OR = 1.80, 95% CI 1.11–2.89), with an even stronger association in males. No link was found with tumor stage. The findings suggest that the *UGT2B7* variant may increase susceptibility to proximal CRC, particularly among men [137].

### 4.7. UGT2B and Papillary Thyroid Cancer

Kilfoy et al. investigated whether genetic polymorphisms in metabolism and detoxification genes influence the risk of papillary thyroid cancer (PTC) and whether these associations are modified by alcohol or tobacco use. A total of 1647 SNPs in 132 candidate genes was analyzed among 344 PTC cases and 452 controls [138]. While nine SNPs and seven gene regions initially showed associations with PTC risk, none remained significant after correction for multiple testing. However, significant gene–environment interactions were observed: *UGT2B7* and NAT1 polymorphisms interacted with alcohol intake, and *CYP26B1* polymorphisms interacted with tobacco use. These findings suggest that detoxification gene variants may modify the effects of alcohol and tobacco on PTC risk, though larger studies are needed to confirm these preliminary observations [138].

### 4.8. UGT2B and Endometrial Cancer

The progression of endometrial cancer (EC) is closely linked to estrogen levels, with UGTs serving as key phase II enzymes involved in steroid hormone detoxification. The role of UGTs in estrogen metabolism and the development of EC has been explored [139]. A total of 100 EC patients and 100 healthy controls was analyzed for *UGT* gene polymorphisms and estrogen levels, while UGT expression was examined in tumor and adjacent tissues from six EC cases. Results revealed disrupted estrogen homeostasis in EC patients, marked by elevated carcinogenic catechol estrogens (4OHE1, 2OHE1, 2OHE2) and reduced estrogen glucuronides [139]. Moreover, *UGT1A8* and *UGT2B7* expressions were significantly downregulated in EC tissues, and the genotype distributions of *UGT1A8* rs1042597 and *UGT2B7* rs7439366 differed notably between patients and controls [139]. The allele frequency analysis revealed that the C allele was more prevalent in EC patients (59%) compared to healthy controls (43.5%), whereas the T allele was more frequent in the control group (56.5%) than in EC patients (41%) [139]. These findings suggest that altered UGT1A8 and *UGT2B7* activity and genetic variation may impair estrogen detoxification and play a critical role in the pathogenesis of EC.

### 4.9. UGT2B and Ovarian Cancer

Grant et al. investigated the associations between genetic variants in the vitamin D receptor (VDR) and related pathway genes with epithelial ovarian cancer (EOC) among women of African ancestry [140]. Using a custom 533,631-SNP Illumina OncoArray and imputation from the 1000 Genomes reference, data from 755 EOC cases (including 537 high-grade serous ovarian cancer-HGSOC) and 1235 controls were analyzed. Significant associations were found in the UGT2A1/2 region with EOC and in both EGFR and UGT2A1/2 with HGSOC. SNPs of *UGT2B4* and *UGT2B10* were also associated with EOC [140].

A summary of genetic association studies evaluating variants in *UGT2B* with cancer susceptibility across different populations is outlined in Table 2, and their relevance to the treatment of cancers is outlined in Table 3. The frequency of mutations/variants among different cancers, and their cooccurrence tendencies is depicted in Supplementary Figure S1 and Table S2. The functional roles of *UGT2B* in hepatocytes and cancer cells as well as their role in steroid hormone metabolism are depicted in Figure 1 and Figure 2.

**Table 2:** Summary of genetic association studies evaluating variants in *UGT2B* with cancer susceptibility across different populations.

	Authors	Country; Population/Ethnicity	Cancer Type	Study Design	Sample Size (Cases/Controls)	Enzymes/Gene Variant/RS IDs Studied	Genotyping Method	Adjustment for Confounders (Age, Smoking, BMI, Family History)	Inferences
1	MacLeod et al. 2000 [100]	USA; African American and Caucasian	PCa	Case–control study	64/64	*UGT2B15* D85Y polymorphism	Allele-specific polymerase chain reaction (PCR) method	Controls matched by age, race, and residence country	Prostate cancer patients showed a significant excess of homozygosity for the D85 allele
2	Gsur et al. 2002 [110]	Austria; Caucasian	PCa	Case–control study (Controls: BPH patients)	190/190	*UGT2B15*(D85Y)	Oligonucleotide ligation assay	Controls matched on age	No association observed between *UGT2B15*(D85Y) polymorphism and PCa risk
3	Hajdinjak et al. 2004 [101]	Slovenia; Slovenian Caucasian	PCa	Association study (Case–control)	206/178 (Controls: women blood donors)	*UGT2B15* D85Y	RFLP assay	Correlation confirmed after age adjustment.	D85Y polymorphism correlates with differentiation of PCa. Comparing controls to patients with Gleason score 7 and above, DD homozygosity frequency difference was significant
4	Park et al. 2004 [102]	USA (H. Lee Moffitt Cancer Center); White men	PCa	Hospital-based Case–control study (age-matched)	155/155	*UGT2B15* Asp85Tyr (D85Y) polymorphism	Allelic-specific PCR analysis	Adjusted for age, alcohol consumption, and smoking pack-years.	The *UGT2B15* Asp85/Asp85 (D/D) genotype increased risk; Overall (Asp/Asp vs. Tyr/Tyr ref)
5	Sparks et al. 2004 [121]	USA (Western Washington State); Caucasian and Asian women	BC (Risk of ER- or PR- tumor/Hormone concentrations)	Prospective cohort study (Cross-sectional analysis of cancer cases)	163 Cases	*UGT1A1* ((TA)6/(TA)7); *UGT2B4* (Asp458Glu); *UGT2B7* (His268Tyr); *UGT2B15* (Asp85Tyr);	oligonucleotide ligation assay	Logistic regression adjusted for age, menopausal status, BMI, smoking, and parity. Hormone analyses adjusted for age, tamoxifen use, BMI, smoking, alcohol use, and race.	*UGT2B15* (Hom. variant Asp85Tyr vs. Wild-type ref) associated with reduced risk of ER-tumor
6	Lin et al. 2005 [132]	China (Shanghai); Chinese Han (Benzidine-exposed workers)	BCa	Case–control study (Cohort comparison)	36/218 (Controls: healthy general population)	*UGT2B7* 802C>T (His268Tyr)	PCR-based procedure	None explicitly stated in OR calculations.	T/T genotype carriers at elevated bladder cancer risk compared to healthy controls
7	Okugi et al. 2006 [103]	Japan; Japanese population (Familial PCa)	PCa	Case–control study	102/117 (Controls: healthy age-matched males)	*UGT2B15*, CAG repeat length of androgen receptor (AR), *CYP17*, 5 alpha reductase type II (SRD5A2), PSA promoter genes	PCR-based RFLP method	Controls age-matched; adjusted in logistic regression.	Analysis showed DD genotype significantly increased PC risk. Presence of Y allele (D/Y + Y/Y vs. D/D ref) was protective. The combination of *UGT2B15* and *CYP17* identified higher-risk individuals
8	Park et al. 2006 [104]	USA (Florida, Arkansas); Caucasian and African American men	PCa	Case–control study	420/487 (293 Caucasians cases/367 controls; 127 AA cases/120 controls)	*UGT2B17* deletion polymorphism (CNV)	PCR analysis	Adjusted for race, age, and pack-years.	*UGT2B17* null genotype significantly increased risk, primarily in Caucasians
9	Gallagher et al. 2007 [113]	USA (Arkansas); Caucasian	PCa	Case–control study	411/397	*UGT2B17* gene deletion polymorphism (CNV)	High-throughput real-time PCR with allelic discrimination	Adjusted for age (continuous), smoking pack-years (continuous), and family history of prostate cancer (categorical).	No association detected between the *UGT2B17* gene deletion polymorphism and prostate cancer risk in Caucasians
10	Karypidis et al. 2008 [105]	Sweden; Caucasian men	PCa	Population-based case–control study	176/161	*UGT2B17* deletion polymorphism (del/ins, del/del)	Real-time PCR (used for gene expression analysis, genotyping method for CNV not explicitly stated)	Adjusted for age.	Deletion carriers showed increased risk (Del/ins + Del/del vs. Ins/ins ref)
11	Park et al. 2007 [106]	USA (Tampa, FL); White men (primarily)	PCa	Hospital-based Case–control study	356/363 (primarily White)	*UGT2B17* null polymorphism; HSD3B1 367N>T	Dideoxy DNA sequencing (examining PCR-amplified DNA)	Adjusted for age and family history of prostate cancer.	*UGT2B17* null genotype was associated with increased PC risk; Combined *UGT2B17* null with HSD3B1 367Asn/Asn was also determined to high risk
12	Zimmermann et al. 2008 [133]	Germany; Caucasian	Bca	Case–control study (Clinical cases vs. Urological controls)	211/210	*UGT2B7* c.802C>T (His268Tyr)	PCR-RFLP procedure	Stratified by smoking status; Mantel–Haenszel estimates adjusted for smoking.	No relevant association observed between *UGT2B7* genotypes and increased bladder cancer occurrence in Caucasians; T/T carriers (vs. C/C ref)
13	Olsson et al. 2008 [111]	Sweden; Swedish men (CAPS study)	PCa	Population-based case–control study followed for mortality	2779 Cases/1722 Controls	*UGT2B17* deletion (Del/Del genotype)	Not explicitly detailed in excerpt	Matched by age and geographical region.	No association found with PC risk or PC death (Del/Del vs. Insertion ref)
14	Van der Logt et al. 2009 [137]	Netherlands; Caucasian (Dutch)	CRC (Sporadic)	Case–control study	411/600	*UGT2B7* H268Y polymorphism (*1 and *2 alleles)	Dual-color real-time PCR assay	ORs adjusted for age and gender.	No overall difference in genotype distributions
15	Setlur et al. 2010 [112]	Austria; Caucasian (Tyrol screening program)	PCa	Case–control study	221/205	CNV of *UGT2B17* and *UGT2B28.*	Affymetrix Genome-Wide Human SNP Array 6.0.	Adjusted for age	No association between *UGT2B* CNVs and PCA risk. HSD3B1 rs6428830 (AA vs. GG ref) was significantly high risk. Combination of HSD3B1 and SRD5A1 risk alleles also significant
16	Hu et al. 2010 [114]	Australia; Australian population-based (Caucasian)	PCa (Incident cases)	Case–control study	826/731	*UGT2B17*-155G/A SNP, FOXA1 binding site (promoter)	Multiplex PCR	Regression models adjusted for age and laboratory assay	The *UGT2B7*-155A allele shows higher promoter activity than the -155G allele in prostate cancer (LNCaP) cells; the -155G allele weakens FOXA1 binding, reducing promoter stimulation and expression; despite affecting androgen metabolism, the –155G/A polymorphism shows no association with prostate cancer risk
17	Haakensen et al. 2010 [123]	Norway/USA (Oslo); Women (Caucasian)	BC (Mammographic Density correlates)	Gene expression analysis of breast biopsies (Cohort with cross-sectional analysis)	64 Cancer Patients/79 Healthy Controls	*UGT2B7, UGT2B10* (rs1828705), *UGT2B11* (Expression/SNPs)	GWAS (Illumina Human-1 109K BeadChip); candidate gene study (iPlex, Sequenom MassARRAY)	Linear regression predicting MD adjusted for age (forced), BMI, current hormone therapy, age at first birth, and parity.	*UGT2B10* expression was a significant, independent predictor of MD in women influenced by female hormones. Down-regulation of *UGT2B7, UGT2B10, UGT2B11* expression associated with higher MD.
18	Nadeau et al. 2011 [107]	Canada (Quebec) and Asia; Caucasian and Asian men	PCa (Biochemical Recurrence—BCR)	Cohort study (evaluating outcomes after prostatectomy)	846 total men (526 Caucasians; 320 Asians)	CNV of *UGT2B17, UGT2B28*	Not explicitly specified in excerpt (CNV analysis performed)	Multivariate Cox regression adjusted for age, PSA, Gleason score, T stage, Nodal status, hormonotherapy, and *UGT2B17/UGT2B28* status.	Deletion copies of *UGT2B* genes increased the risk of PSA recurrence (BCR) in Caucasians (1 deletion vs. 0 ref). In Asians *UGT2B17* (2 deletions vs. ≤1 deletion ref) posed significant risk. Combined *UGT2B17 + UGT2B28* (≥2 deletions vs. ≤1 deletion ref) posed risk in both Caucasians and Asians.
19	Sun et al. 2011 [118]	USA, Barbados, Nigeria; African ancestry	BC	Case–control study	1261/825	*UGT2B4* tag SNP rs13129471 (upstream region)	Custom Taqman assay	Not explicitly listed in excerpt for odds ratio calculation	SNP rs13129471 (A allele) associated with increased breast cancer risk; overall (Heterozygote vs. G/G ref)
20	Parmar et al. 2011 [141]	Austria (TIGER cohort)	BC	Pharmacogenetic study (PGt) based on a retrospective cohort	745 total BC patients (205 epirubicin-treated)	*UGT2B7* His268Tyr polymorphism (802 C>T)	Validated TaqMan^®^ SNP Genotyping Assay via Real-time PCR	Tumor size, age at diagnosis, nodal status, histological grade	BC patients carrying the *UGT2B7* (Tyr/Tyr) genotype may derive the greatest benefit from adjuvant epirubicin-based chemotherapy, demonstrating longer invasive disease-free survival. This effect was more pronounced when subsequent tamoxifen treatment was administered.
21	Sun et al. 2012 [142]	USA, Barbados, Nigeria; African ancestry	BC	Case–control study (Combined 3 populations)	1287/851	*UGT2B15* D85Y (rs1902023); *UGT2B15* T523K (rs4148269); *UGT2B7* H268Y (rs7439366)	Commercial TaqMan assays	Adjusted for population indicator variable in logistic regression.	Lack of association between common *UGT2B* nonsynonymous SNPs and breast cancer in populations of African ancestry
22	Aschebrook-Kilfoy et al. 2012 [138]	USA; European ancestry	Papillary Thyroid Cancer (PTC)	Nested case–control study within a cohort (USRT) + hospital cases (UTMDACC)	344/452	1647 tagging SNPs in 132 genes/regions; *UGT2B7* SNPs (rs7657426, rs4986993, rs9650592, rs7606254, rs194243, rs7387059, rs7837181, rs3924192, rs3924194, rs975612, rs11681809, rs15561)	Custom-designed iSelect Infinium assay (Tagging SNPs)	Adjusted for gender, age, and year of birth. Interaction analyses adjusted for alcohol or tobacco use.	*UGT2B7*, SOD1, CYP8B1, MTF2, GSTT1, DHRS9 and FMO3 were associated with PTC. All of genetic regions not significant after multiple comparison correction.
23	Dura et al. 2012 [131]	Netherlands; Caucasian	Esophageal Cancer (ESCC, EAC)	Case–control study	351 (85 ESCC, 260 EAC)/592	*UGT1A1; UGT1A6, UGT1A7, UGT1A8; UGT2B4; UGT2B7; UGT2B17* (Enzyme activity/Genotype groups)	Various PCR-based assays (e.g., PCR-RFLP, real-time PCR)	Matched for age, ethnicity, gender. Stratified by histology.	*UGT2B4* high- and medium-activity genotypes are protective against ESCC; *UGT1A8* high-activity genotype decreased ESCC risk; *UGT1A1* high-activity genotype increased ESCC risk; haplotype *UGT2B7 + UGT2B17* mutation (011) reduced ESCC risk
24	Eskandari-Nasab et al. 2012 [124]	Iran; Iranian population	BC	Case–control study	236/203 (expression assay in 62 breast cancerous and 62 adjacent noncancerous tissue)	*UGT2B17* null genotype (CNV);	multiplex PCR assay; PCR allele-specific amplification; quantitative reverse transcriptase PCR	Logistic regression adjusted for age	*UGT2B17* null genotype significantly increased cancer risk; DHFR 19 bp ins/del polymorphism showed no association; NGX6 mRNA levels were significantly lower in cancerous tissue
25	Vidal et al. 2013 [108]	USA; African American and Caucasian (Multiethnic)	PCa	Hospital-based case–control study	233/342	*UGT2B15* (rs4148269, rs3100, rs9994887, rs13112099, rs7686914, rs7696472, rs1580083, rs1960773); *UGT2B17* (rs7435827, rs7686008, rs7671342, rs59678213); Cis acting at *UGT2B15* and *UGT2B17* (rs17147338, rs2168047); Cis acting at *UGT2B17* (rs6822259)	Sequenom-iPlex Genotyping	Adjusted for age, race, and BMI.	Six *UGT2B15* SNPs (rs4148269, rs3100, rs9994887, rs7686914, rs7696472, and rs13112099) and a cis acting (rs17147338) associated with increased PC risk
26	Angstadt et al. 2013 [134]	USA (Pennsylvania); Caucasian	CRC	Population-based case–control study	665/621	*UGT2B17*, *UGT2B28* (CNVs)	TaqMan Copy Number Assays (Custom and Predesigned) using 7900-HT real-time PCR in quadruplicate.	Multivariate models adjusted for age, sex, BMI, first degree family history, NSAID use, and physical activity.	*UGT2B17* (0/0) genotype showed decreased overall CRC risk, specifically in rectal cancer and in males. No association found for *UGT2B28*.
27	Gruber et al. 2013 [125]	Austria; Austrian Caucasian	Lung Cancer	Retrospective case–control study	453/449	*UGT2B17* (CNV)	Conventional PCR followed by sequencing verification	Adjusted for gender, smoking status, and histologic subtype.	No significant association observed between *UGT2B17* CNV and lung cancer risk or outcome
28	Grant et al. 2013 [109]	USA (Durham, NC); Black and White men	Pca	Hospital-based case–control study	*UGT2B15*D85Y: 92 Cases/297 Controls; *UGT2B17* CNV: 148 Cases/201 Controls	*UGT2B17* CNV, rs7434408; *UGT2B15* D85Y (rs1902023); *UGT2B7* (rs7435335)	Sequenom-iPlex	Adjusted for age and race.	*UGT2B15* D85/D85 (G/G) homozygosity associated with increased risk (G/G vs. T/T ref); *UGT2B17* CNV, two-copy genotype associated with higher androstane-3a,17b-diol-glucuronide levels in Whites but not in Blacks
29	Vulsteke et al. 2013 [143]	Leuven, Belgium	BC	Retrospective cohort study	1012 female breast cancer cases who received 3–6 cycles of FEC as neoadjuvant or adjuvant therapy	Twenty-six SNPs in 16 genes, including *UGT2B7* (rs7668282)	Sequenom MassARRAY	Primary growth factor use, age, BMI, and number of planned cycles	*UGT2B7* rs7668282 C-allele carriers were also associated with prolonged grade 4 or deep neutropenia
30	Scherer et al. 2014 [144]	Multi-site (CCFR); Caucasian	CRC	Matched case–sibling control study (CCFR)	1584/2516 (Unaffected sibling controls)	Total of 35 functional polymorphisms including UGT familz member genes *UGT2B15; UGT2B4; UGT1A3; UGT1A6*	TaqMan assays	Adjusted for age, sex; BMI, pack-years, and physical activity were also adjusted for NSAID interaction	*UGT2B15*, TG heterozygotes and G alleles had increased CRC risk (vsTT); individuals homozygous for the *UGT2B15* minor allele, who used aspirin, had higher CRC risk (vs T/T non-users); Ibuprofen users homozygous for *UGT2B4* major alleles had increased risk (vs. major alleles homozygous, non-users)
31	Angstadt et al. 2014 [145]	USA (Pennsylvania); Caucasian	CRC	Population-based case–control study	857/932 (*UGT2B* analysis)	9 *UGT1A* and 5 *UGT2B* genes. Analysis included 85 SNPs in *UGT1A* and 12 SNPs along with one deletion/insertion polymorphism in *UGT2B*	Illumina GoldenGate genotyping assay	Multivariate models adjusted for age, sex, BMI, family history, NSAID use, and physical activity.	*UGT1A10* exon 1 (T-G haplotype; rs17864678, rs10929251) decreased colon cancer risk both proximal and distal; *UGT1A* 3′ flanking region (C-T-G haplotype; rs7578153; rs10203853; rs6728940) increased CRC risk in males; *UGT2B15* haplotype (rs4148269, K523T + rs6837575) increased rectal cancer risk overall and in females; *UGT1A* shared exon haplotype (A-G-T; rs6717546, rs1500482, rs7586006) with high NSAID use and decreased CRC risk
32	Hu et al. 2015 [15]	Southwest China; Chinese Han	Esophageal Squamous Cell Carcinoma (ESCC)	Case–control study investigating Copy Number Variations (CNVs)	404/402	CNV of *UGT2B28, UGT2B17*, and other genes e.g., PLEC	Custom Multiplex AccuCopy Kit	Adjusted for age, gender, smoking/drinking status, and family cancer history.	Copy number loss of *UGT2B28* conferred increased ESCC risk (*UGT2B28* Deletion carriers vs. 2 copies as ref) associated with decreased *UGT2B28* mRNA expression in tumor tissues; PLEC copy number gain also increased ESCC risk; Concordant germline and somatic CNV alterations observed for PLEC, *UGT2B17*, and *UGT2B28,* but not for other loci
33	Habibi et al. 2017 [115]	Iran; Iranian population	PCa and Benign Prostatic Hyperplasia (BPH)	Case–control study (PC vs. BPH vs. Controls)	120 (PC)/120 (Healthy Controls)/120 (BPH)	*UGT2B15* D85Y (rs1902023); CNV of *UGT2B17* and *UGT2B28* loci	PCR-RFLP for *UGT2B15* D85Y	Questionnaire covered smoking, BMI, PSA level (no explicit adjustment used in ORs provided).	No association found between *UGT2B15* D85Y, *UGT2B17* CNV, or *UGT2B28* CNV and PC risk. *UGT2B17* deletion genotypes significantly more frequent in BPH vs. healthy controls
34	Grant et al. 2017 [68]	USA (Duke University/VA Medical Center); Black (48%) and Non-black (52%) men	PCa (Biochemical Recurrence—BCR)	Cohort study (evaluation post-prostatectomy)	190 total patients	*UGT2B15, UGT2B17,* and *UGT2B28* (Expression levels, measured by percent positive cells and H-score)	Immunohistochemical detection (IHC)	Adjusted for PSA, age, pathological Gleason score, race, positive surgical margins, extracapsular extension, and seminal vesicle invasion (Cox model).	*UGT2B17* overexpression (High vs. Low ref) was associated with BCR risk. Crude HR = 1.638 (95% CI 1.050, 2.557). Adjusted HR = 1.547 (95% CI 0.985, 2.433)
35	AL-Eitan et al. 2019 [120]	Jordan; Jordanian–Arab population	BC	Case–control study	218/219	*UGT2B7* (rs28365062, rs4348159);	Not explicitly stated	Modified Bonferroni procedure applied. Crude ORs provided.	No significant association found between the investigated *UGT2B7* SNPs and BC risk. rs4348159 (TT vs. CC ref): OR = 1.08 (95% CI 0.41–2.88).
36	He et al. 2018 [119]	China (Zhengzhou); Chinese Han	BC	Retrospective case–control study	672/670	*UGT2B7* tagSNPs (rs12233719, rs4356975, rs7435335, rs7441774)	MALDI-TOF MS	Adjusted for conventional risk factors (e.g., age, BMI, menopausal status, family history) via multiple logistic regression.	*UGT2B7* rs7441774 G allele associated with increased breast cancer risk. GG genotype: adjusted OR = 1.63 (95% CI 1.18–2.26)
37	Grant et al. 2019 [140]	Multi-site (AACES, OCAC); African Ancestry (AA) women	Epithelial Ovarian Cancer (EOC) and High-Grade Serous Ovarian Cancer (HGSOC)	Combined case–control study (pooled from consortia: GAME-ON project)	EOC: 755/1235 (Total *N* = 1990). HGSOC: 537/1235 (Total *N* = 1772).	Several SNPs in VDR, *UGT2B, UGT1A, UGT2A1/2*,22 SNPs in *UGT2B4* and 1 SNP of *UGT2B10*	Illumina OncoArray (533,631 SNP array), followed by imputation (Minimac3) to 1000 Genomes Phase 3 v5 reference set.	Adjusted for two principal components (PCs) of ancestry using logistic regression.	African American OncoArray analysis showed SNPs of *UGT2B4* and *UGT2B10* associated with EOC. Bayesian False Discovery Probability analysis confirmed significant SNP associations with epithelial ovarian cancer (EOC) in the UGT2A1/2 region (rs10017134) and with high-grade serous ovarian cancer (HGSOC) in the EGFR (rs114972508) and UGT2A1/2 (rs1017134) regions
38	Qian et al. 2021 [126]	China (Shenyang, Tianjin); Never-smoking Chinese women (Han ethnic)	Non-small cell lung cancer (NSCLC)	Two-stage, case–control study	Training: 417/368; Validation: 282/282	*UGT2B7* rs12233719 (G>T); *UGT2B7* rs7439366	TaqMan method	Multivariate logistic regression adjusted by age and family history of cancer.	*UGT2B7* rs12233719 T allele associated with increased NSCLC risk
39	Zhao et al. 2020 [139]	China (Xuzhou Central Hospital); (likely Chinese Han)	Endometrial Cancer (EC)	Case–control study	100 EC patients/100 healthy subjects	*UGT1A8* rs1042597; *UGT2B7* rs7439366	PCR followed by ABI sequencing.	Subjects were excluded based on narrow BMI range (18.5–24.99), menarche age (12–16 y), and smoking history. Age difference was assessed and found non-significant.	The distribution of genotypes for both SNPs was significantly different between cases and controls. *UGT1A8* rs1042597: Genotype frequencies (CC, CG, GG) significantly differed. *UGT2B7* rs7439366: Genotype frequencies (CC, CT, TT) significantly differed. Allele frequencies (C/T) significantly differed (C allele higher in cases, 59% vs. 43.5%). The study infers that these polymorphisms may influence altered circulating hormones and EC risk

PCa, prostate cancer; BC, breast cancer; BCa, bladder cancer; CRC, colorectal cancer; PTC, papillary thyroid cancer; ESCC, esophageal squamous cell carcinoma; EC, esophageal cancer; BPH, benign prostatic hyperplasia; EOC, epithelial ovarian cancer; HGSOC, high-grade serous ovarian cancer; BCR, biochemical recurrence; ER-/PR-, estrogen/progesterone receptor negative; MD, mammographic density.

**Table 3:** Overview of pharmacogenetic studies investigating the influence of *UGT2B* polymorphisms on chemotherapy treatment outcomes.

	Authors	Country; Population/Ethnicity	Cancer Type	Study Design	Sample Size (Cases/Controls)	Enzymes/Gene Variant/RS IDs Studied	Genotyping Method	Adjustment for Confounders (Age, Smoking, BMI, Family History)	Inferences
1	Parmar et al. 2011 [141]	Austria; Austrian Caucasian (TIGER cohort)	BC (Adjuvant treatment outcome)	Pharmacogenetic cohort study	745 total patients (205 received epirubicin)	*UGT2B7* His268Tyr (c.802 C>T)	TaqMan assays (Real Time PCR System 7300)	Adjusted for tumor size, age at diagnosis, nodal status, and histological grade (Cox model).	The *UGT2B7* His268Tyr (802 C>T) was associated with shorter invasive disease-free survival after epirubicin treatment. (Tyr/Tyr vs. His allele carriers ref)
2	Ahern et al. 2011 [146]	Denmark (Jutland Peninsula); Caucasian women	BC (Recurrence in Tamoxifen-treated survivors)	Nested case–control study within a population-based cohort	ER+/TAM+ Group: 541/541; ER−/TAM− Group: 300/300	UGT2B15*2 (rs1902023, 85 D>Y); UGT2B7*2 (rs7439366, 268 H>Y); UGT1A8*3 (rs17863762, 277 C>Y)	Applied Biosystems kits (C-27028164-10, C-34418788-20) and custom TaqMan kit for proxy SNP (rs7434332)	Adjusted for tumor stage, menopausal status, adjuvant systemic chemotherapy/radiotherapy, type of surgery, time to recurrence, and histologic grade.	No association found between UGT polymorphisms and breast cancer recurrence risk in either tamoxifen-treated or non-tamoxifen-treated groups; UGT2B15*2 (Two variant alleles vs. 2 normal alleles ref)
3	Sawyer et al. 2016 [147]	Not specified (Multi-center study)	Early stage BC receiving epirubicin (FEC regimen)	Prospective pharmacogenetic study	132 women enrolled (26 CC, 63 CT, 37 TT)	*UGT2B7*c.-161 C>T germline SNP (rs7668258)	PSQ 96 HSA genotyping system (pyrosequencing)	Not available	The CC genotype showed significantly reduced epirubicin clearance compared with those with CT or TT genotypes. Also, CC homozygotes were at an increased risk of grade 3 to 4 leukopenia compared with TT homozygotes or heterozygotes; TT homozygotes or heterozygotes had an increased risk of early recurrence
4	Li et al. 2019 [148]	Wuhan, China (Chinese BC patients)	BC	Prospective cohort study	427 BC patients (141 CC, 196 CT, 90 TT)	*UGT2B7*-161 single-nucleotide polymorphism (C>T, rs7668258)	PCR amplification followed by pyrosequencing on the PSQTM96MA System	Age, BMI, smoking, hypertension, TNM stage, cumulative dose of epirubicin, administration of trastuzumab, cTnI, NT-proBNP	The *UGT2B7*-161 T allele independently predicts a low occurrence of cardiotoxicity in BC patients undergoing EC-D adjuvant chemotherapy. Cardiotoxicity rate was lowest in the TT group (1.1%).
5	Joy et al. 2021 [149]	Canada (Caucasian 87%)	Early stage BC	Prospective dose-tailoring study (intrapatient dose escalation trial)	45 early-stage BC patients (10 CC, 21 CT, 14 TT genotypes)	*UGT2B7*-161 promoter polymorphism (C>T, rs7668258)	PSQ 96 HSA genotyping system (Pyrosequencing AB)	None specified for primary toxicity/survival, though PK covariates such as body weight, height, body surface area, lean body mass, age, and liver function are considered in the model	Pharmacogenetically guided epirubicin dosing (escalating doses for CT/TT genotypes) is feasible and well-tolerated, as leukopenia rates remained comparable across all genotypes after dose escalation.
6	Jian Li et al. 2022 [150]	Shenzhen, China (Chinese HER-2 positive BC patients)	HER-2 positive BC	Cohort study/Exploratory study	50 patients (24 CC, 15 CT, 11 TT) and 30 healthy controls	*UGT2B7*-161 single-nucleotide polymorphism (rs7668258)	PCR kit and the 7900HT PCR instrument; ABI 3730XL sequencer	Logistic Multivariate Regression included *UGT2B7*-161 genotypes, BMI, hyperurecemia and cardiac troponin I (cTnI)	The *UGT2B7*-161 SNP is a potential predictive factor for cardiotoxicity (assessed by myocardial work decreased events using PSL) in HER-2 positive BC patients receiving dual-targeted therapy. The CC genotype had a significantly higher incidence of myocardial work decrease events (41.7%) compared to CT + TT (12.5%). The BMI, and cTnI were also other independent factors affecting the occurrence of myocardial toxicity
7	Kruger et al. 2026 [59]	South Africa; Women of Mixed and African Ancestry receiving tamoxifen	BC	Observational pharmacogenetic association study in tamoxifen-treated cohort	166 breast cancer patients (no separate controls reported)	*UGT2B7:* rs7439366 *UGT2B15:* rs4148269	Mass spectrometry-based genotyping assay Sanger DNA sequencing Polymerase chain reaction (PCR)-based genotyping TaqMan real-time PCR copy number assay Copy number variation (CNV) genotyping assay Real-time quantitative PCR (qPCR) platform	Logistic regression and Bonferroni correction	. Musculoskeletal complaints associated with *UGT2B7* rs7439366 and CYP3A4 rs2242480. Gynecological symptoms associated with *SULT1A*2*2, *SULT1E1* rs3736599, and *UGT2B15* rs4148269. Hot flashes showed no significant pharmacogenetic association. Findings support the relevance of pharmacogenetic variability in tamoxifen tolerability in African populations.

BC, breast cancer; EC, endometrial cancer; FEC, 5-Fluorouracil + Epirubicin + Cyclophosphamide.

## 5. *UGT2B* Polymorphisms Clinical Implications and Pharmacogenomics

Scherer et al. examined whether genetic variations in NSAID-metabolizing enzymes, particularly *CYP2C9* and *UGT* genes, affect the risk of colorectal cancer (CRC) and modify the protective effect of NSAIDs. Using data from 1584 CRC cases and 2516 sibling controls in the Colon Cancer Family Registry, researchers analyzed 35 functional polymorphisms [144]. They found that specific variants *UGT1A6* GAA (Ala7Thr181Arg184) and *UGT2B15* Asp85 were associated with increased CRC risk. Additionally, significant gene–NSAID interactions were identified, including between *UGT1A3* Thr78Thr and NSAID use, *UGT2B4* variants and ibuprofen use, and *UGT2B15* Tyr85Asp and aspirin use. These findings suggest that UGT polymorphisms may influence both CRC susceptibility and the chemopreventive efficacy of NSAIDs, underscoring the importance of pharmacogenetic profiling in CRC prevention strategies [144].

Ning et al. examined the impact of *UGT2B7* gene polymorphisms on the efficacy of morphine for cancer pain in 120 Chinese Han patients receiving morphine via patient controlled analgesia [151]. Pain intensity was measured using the visual analog scale (VAS) at multiple time points up to 72 h, and plasma morphine levels were determined alongside genotyping for *UGT2B7* c.802C>T and c.221G>T variants. The c.802C>T polymorphism showed significant influence: patients with CT or TT genotypes had higher VAS scores (indicating poorer analgesic response) and higher plasma morphine concentrations than those with the CC genotype. No significant differences were observed for c.221G>T genotypes. These findings suggest that the *UGT2B7* c.802C>T variant, but not c.221G>T, affects morphine metabolism and analgesic efficacy in Chinese Han patients with cancer pain [151].

Several studies have assessed the role of *UGT2B* polymorphisms in patients with breast cancer receiving epirubicin-based chemotherapy, providing evidence for the potential role of pharmacogenomics in improving treatment safety/outcomes. A prospective study examined the impact of the *UGT2B7* c.161C>T SNP on epirubicin treatment outcomes in women with non-metastatic breast cancer receiving chemotherapy [147]. Patients with the CC genotype showed significantly lower epirubicin clearance and had a higher risk of severe leukopenia (grade 3–4) compared to CT and TT carriers. Conversely, TT and CT genotypes were associated with a higher risk of early cancer recurrence [147]. These findings suggest that the *UGT2B7* c.161 C>T polymorphism influences both drug toxicity and efficacy, supporting its potential as a pharmacogenetic marker for tailoring epirubicin-based treatment. The effect of *UGT2B7* His268Tyr polymorphism in 745 non-metastatic breast cancer patients treated with epirubicin-based chemotherapy was also studied [141]. Among the 205 epirubicin-treated patients, those with the Tyr/Tyr genotype had significantly longer invasive disease-free survival compared to carriers of at least one His allele. The survival benefit was even greater in patients who also received tamoxifen [141]. No survival difference was observed in patients not treated with epirubicin. These findings suggest that the *UGT2B7* 268Tyr/Tyr genotype may predict better outcomes with epirubicin and support its potential as a biomarker for personalized breast cancer therapy. Similarly, Parmar et al. investigated the impact of the *UGT2B7* His268Tyr polymorphism on invasive disease-free survival (IDFS) in 745 non-metastatic breast cancer patients from the Austrian TIGER cohort. Among 205 patients treated with epirubicin-based chemotherapy, those homozygous for the 268Tyr allele (Tyr/Tyr) had a longer mean IDFS of 8.6 years compared to 7.5 years in patients carrying at least one 268His allele (adjusted hazard ratio [HR] = 2.64; *p* = 0.014) [141]. The effect was more pronounced in patients subsequently treated with tamoxifen (adjusted HR = 5.22; *p* = 0.015) [141]. No significant differences in IDFS were observed in patients who did not receive epirubicin [141].

Vulsteke et al. showed that variants in *UGT2B7* (C-allele carriers of the rs7668282) were associated with febrile neutropenia (FN) in breast cancer cases receiving FEC regimen. However, after correcting for multiple testing (FDR), only rs4148350 (T allele) in ABCC1 remained significantly associated with FN. Nevertheless, *UGT2B7* (C-allele carriers of the rs7668282) was associated with prolonged grade 4 neutropenia, which persisted significantly after multiple testing corrections [143].

Joy et al. evaluated whether *UGT2B7*c.-161 C>T promoter polymorphism carriers could safely tolerate higher epirubicin doses. Forty-five women with non-metastatic breast cancer received standard FE100C in the first cycle, followed by genotype-based dose escalation in subsequent cycles (up to 130 mg/m^2^ for CT and 140 mg/m^2^ for TT patients). Leukopenia incidence increased with dose in CT and TT groups but remained comparable to CC patients at standard doses [149]. The findings suggest that genotype-guided epirubicin dosing enables safe dose escalation without added toxicity [149]. Similarly, Li et al. evaluated the relationship between the *UGT2B7*-161 C>T polymorphism and cardiotoxicity in 427 Chinese breast cancer patients receiving epirubicin/cyclophosphamide-docetaxel (EC-D) adjuvant chemotherapy. Patients were genotyped as CC (*n* = 141), CT (*n* = 196), and TT (*n* = 90). Cardiotoxicity was defined by a ≥10% decline in left ventricular ejection fraction (LVEF) to <53%, heart failure, acute coronary syndrome, or fatal arrhythmia [148]. LVEF decreased significantly during and up to 12 months after chemotherapy (*p* < 0.001). Cardiotoxicity occurred in 4.2% of patients overall and was lowest in the TT group (1.1%), compared to CT (3.1%) and CC (7.8%) (*p* = 0.026) [148]. Multivariate analysis indicated that the T allele independently predicted lower cardiotoxicity (*p* = 0.004) [148]. This observation was supported by the investigation by Li et al. among 50 Chinese HER-2 positive breast cancer patients receiving trastuzumab and pertuzumab therapy [150]. Blood samples were collected at baseline to determine *UGT2B7*c.-161 genotypes, and myocardial function parameters were measured before treatment and after four therapy cycles. Among the patients, 35 experienced decreased myocardial work, with occurrences varying by genotype: CC 41.7%, CT 12.5%, and TT 12.5% (*p* < 0.001) [150]. Multivariate analysis identified *UGT2B7*-161 genotype, body mass index, and cardiac troponin I as independent predictors of cardiotoxicity.

Functional polymorphisms in genes coding for UGT enzymes (UGT2B152, UGT2B72, UGT1A8*3) may also affect breast cancer recurrence in women treated with tamoxifen, but no clear evidence is available [146]. Among 541 estrogen receptor-positive (ER+)/tamoxifen-treated and 300 ER-negative/untreated cases (matched with controls), no significant association was found between any UGT polymorphism and recurrence risk [146]. Results remained consistent even when stratified by CYP2D6*4 genotype, another key tamoxifen metabolizing gene [146]. These findings indicate that *UGT* genetic variants do not influence tamoxifen treatment outcomes, and genotyping these UGT polymorphisms is not supported for predicting breast cancer recurrence. A similar study evaluated the impact of genetic variation in *UGT1A4*, *UGT2B7*, and *UGT2B15* on tamoxifen metabolism in Asian breast cancer patients [152]. In 240 healthy individuals from Chinese, Malay, and Indian populations, haplotype tagging SNPs were identified and then tested in 202 tamoxifen-treated patients. The UGT1A4*3 haplotype (142T>G; L48V) was significantly associated with the increased formation of TAMN glucuronide, showing a twofold higher TAMN glucuronide/TAM ratio (*p* < 0.0001). *UGT2B7* variants showed no significant effect on the O-glucuronidation of active metabolites (4OHT, endoxifen), and *UGT2B15* haplotypes had only a modest influence on (E) endoxifen levels after adjusting for *CYP2D6* genotypes [152]. These results highlight the key role of *UGT1A4* polymorphisms in tamoxifen glucuronidation in Asians, with *UGT2B7* and *UGT2B15* variants contributing minimally.

Gene expression of *UGT2B* family members may aid in identifying the non-responders in hormone receptor-negative breast cancer treatment. Gil et al. investigated molecular biomarkers predictive of response to neoadjuvant treatment (NAT) in HER2 positive, hormone receptor (HR)-negative breast cancer [153]. Tissue samples from women who received NAT at several hospitals in Andalusia, Spain, were analyzed. These patients were treated with standard chemotherapy (taxanes and/or anthracyclines) alongside antiHER2 agents such as trastuzumab and pertuzumab and categorized as responders (achieving pathological complete response) or non-responders. Microarray analysis in a discovery cohort (*n* = 20) revealed 954 differentially expressed transcripts. Five genes from the *UGT2B* family, *UGT2B10*, *UGT2B11*, *UGT2B15*, *UGT2B17*, and *UGT2B28,* were selected for validation. qPCR in an independent cohort (*n* = 40) showed overexpression of these genes in non-responders, with *UGT2B15* demonstrating statistically significant association [153]. As *UGT2B* family genes are involved in drug detoxification, *UGT2B15* may influence drug metabolism and predict treatment response, warranting further research.

Few studies have looked at UGT polymorphisms in patients with chronic lymphocytic leukemia (CLL). Analyzing 320 CLL patients and 449 healthy donors, Gruber et al. found that high *UGT2B17* expression was significantly associated with adverse clinical outcomes, such as reduced treatment-free and overall survival in patients with CLL [154]. Elevated mRNA levels correlated closely with increased glucuronidation activity toward androgens and the anticancer drug vorinostat. Notably, *UGT2B17* was upregulated following fludarabine treatment, especially in poor responders. Furthermore, after knocking down *UGT2B17* in MEC1 CLL cells, gene expression profiling revealed significant changes in multiple pathways, indicating that *UGT2B17* actively influences key cellular functions rather than being a passive gene product. Overall, the study identified *UGT2B17* as a functionally relevant biomarker in CLL with potential therapeutic implications [154]. Rouleau et al. explored the regulatory mechanisms underlying the expression of *UGT2B17* [155]. Using RNA sequencing and qPCR, the study found that in both normal and leukemic B cells, *UGT2B17* is expressed exclusively through alternative transcripts distinct from the canonical transcript seen in liver and intestine. Furthermore, genomic and chromatin accessibility data (ATACseq) revealed the presence of alternative promoters, some likely derived from retrotransposons. Functional assays identified key binding sites for transcription factors STAT3, NFκB (RELA), and IRFs, which drive *UGT2B17* expression in CLL cells. These findings suggest that a NFκB/STAT3/IRF/*UGT2B17* axis may play a pivotal role in CLL progression and therapeutic resistance [155]. These factors are activated through key signaling pathways; STAT3 via the JAK pathway, and NFκB/IRFs via B cell receptor (BCR) signaling and Syk activation. Inhibitors targeting these pathways, such as Syk inhibitors (e.g., entospletinib) and JAK inhibitors (e.g., ruxolitinib), can disrupt the activation of these transcription factors. The pharmacological inhibition of STAT3 and NFκB led to reduced *UGT2B17* promoter activity in luciferase assays, suggesting that Syk/JAK inhibition may downregulate *UGT2B17* expression by interfering with its upstream regulatory axis [155].

See Supplementary Table S1 for *UGT2B* gene variant frequencies from populations to populations; this can alter their relevance in predicting not only *UGT2B* family member-mediated treatment outcomes but also disease risks. A genome-wide association study among 2239 smokers from five ethnic groups in the Multiethnic Cohort to examine genetic variants influencing nicotine and cotinine glucuronidation mediated by *UGT2B10* showed association that fifteen key SNPs explained up to 33% of metabolic variation in cotinine glucuronidation, with the strongest association at rs115765562. The other two SNPs showing high significance were *UGT2B10* splice site variant, rs116294140, and rs6175900 (Asp67Tyr). The top SNP for nicotine glucuronidation (rs116224959) showed strong linkage with rs115765562 [156]. Another study examined nicotine metabolism across five ethnic groups, African American, Native Hawaiian, White, Latino, and Japanese American smokers, focusing on the roles of CYP2A6 (C oxidation), *UGT2B10* (N glucuronidation), and FMO3 (N oxidation). Marked ethnic differences were observed: C oxidation was lowest in Japanese Americans and Native Hawaiians, while N glucuronidation was lowest in African Americans [157]. Two *UGT2B10* variants, a missense mutation (Asp67Tyr, rs61750900) and a splice variant (rs116294140), together explained about 33% of the variability in nicotine glucuronidation, with the splice variant being the major contributor to reduced glucuronidation, particularly among African Americans [157]. Overall, findings highlight that genetic and ethnic variation in nicotine metabolism pathways influences smoking behavior and may contribute to ethnic disparities in lung cancer risk.

## 6. *UGT2B* and Cancer-Tissue Studies

Studies have employed primary human tissue, microsomes, or clinical cohort data to establish the oncological relevance and coordinated expression of UGTs [43,158,159,160,161]. Keading et al. demonstrated that calcitriol downregulates *UGT2B15* and *UGT2B17*, which are key enzymes that inactivate androgens in prostate cancer cells (LNCaP and 22Rv1) [78]. Calcitriol treatment led to reduced glucuronidation of dihydrotestosterone (DHT) and its metabolites, along with significant decreases in *UGT2B15*/17 mRNA and protein levels [78]. Since androgens fuel prostate cancer growth, this suppression of androgen inactivation by calcitriol may counteract its intended antiproliferative effects, highlighting a potential limitation in using calcitriol for prostate cancer therapy. Jones and Lazarus (2014) characterized *UGT2B* expression levels in human tissues targeted by tobacco carcinogens [160]. Using quantitative expression analysis across multiple human tissues (e.g., tonsil, lung, liver, aerodigestive tract), the study inferred that extrahepatic *UGT2B* expression is highest in the tonsil, comparable to liver levels. Furthermore, specific *UGT2B* enzymes (e.g., UGTs 2B10, 2B11, 2B17) are expressed and coordinately regulated in these target sites for tobacco-related cancers [160].

Invasive cancers commonly exhibit enhanced aerobic glycolysis and de novo lipid biosynthesis. UDP-glucuronosyltransferases (UGTs), key phase II metabolizing enzymes, normally facilitate the glucuronidation and elimination of lipids; however, their dysregulation in cancer cells may lead to the accumulation of bioactive lipids that promote tumor progression [162]. This study hypothesized that *UGT2B* isoforms are downregulated in cancer cells and that restoring their expression could reduce lipid accumulation, alter cellular phenotype, and suppress proliferation. Steady state mRNA levels of *UGT2B* isoforms were quantified by qPCR in four breast cancer and five pancreatic cancer cell lines. Expression plasmids encoding *UGT2B4*, *UGT2B7*, and *UGT2B15* isoforms involved in lipid glucuronidation were transfected into MCF7 and Panc1 cells [162]. Cell viability and cytotoxicity were assessed using trypan blue exclusion, annexin V/PI staining, TUNEL assays, and caspase3 immunohistochemistry. Overexpression of each *UGT2B* isoform significantly decreased cell proliferation and increased cell death in both cell lines, accompanied by a marked reduction in intracellular lipid levels [162]. These findings support the hypothesis that *UGT2B* enzymes modulate lipid homeostasis and proliferation in cancer cells, highlighting their potential role as “lipid regulators” and therapeutic targets in cancer management.

*UGT2B* member function is not only governed by genetic variants and expression, but also mediated by epigenetic changes mediated by miRNAs. Wijayakumara et al. (2017) described novel miRNA-mediated regulation pathways for *UGT2B4* and *UGT2B7* [161]. Functional assays in HepG2 and Huh7 liver cancer cell lines were complemented by correlative analysis using normal human tissue panels and The Cancer Genome Atlas (TCGA) data. The core inference confirmed that miR3664 controls *UGT2B7*, and miR135a and miR410 control *UGT2B4* expression, which are mechanisms supported by inverse correlations observed in clinical cohorts and tissue panels [161]. The same group also investigated the regulation of *UGT2B15* by miR3315p, focusing on the characterization of its target sites [159]. Experiments in prostate cancer cells, validated using a tissue RNA panel and TCGA data, inferred that miR3315p represses *UGT2B15* activity via the cooperative action of canonical and non-canonical 3′UTR sites. Crucially, this post-transcriptional mechanism differentially regulates *UGT2B15* without affecting *UGT2B17* [159]. Furthermore, Margaillan et al. 2016 analyzed the epigenetic regulation of androgen inactivating *UGT2B15* and *UGT2B17* by microRNAs in prostate cancer progression [158]. Using reporter assays in HEK293 cells and ectopic expression in prostate cancer cells, followed by the analysis of UGT/miRNA levels in prostatic tumors and metastases, the study inferred that miR376c directly downregulates both UGTs. This action reduces androgen inactivation and enhances cell proliferation, and the observed inverse correlation in clinical specimens suggests miR376c critically influences steroid metabolism during cancer progression [158].

Another observational study by Pâquet et al. analyzed *UGT2B15* and *UGT2B17* expression in prostate cancer using tissue samples from a cohort of 239 patients, including 127 prostate cancer cases and 112 benign prostatic hyperplasia (BPH) controls collected at Laval University (Québec) and the University of British Columbia (Vancouver) [163]. mRNA levels were quantified by real-time PCR, and protein expression was assessed via immunohistochemistry. While neither *UGT2B15* nor *UGT2B17* expression correlated significantly with Gleason score, *UGT2B17* was significantly upregulated in all tumor grades and was fivefold higher in metastases compared with benign tissue [163]. In contrast, *UGT2B15* expression was markedly decreased in both treatment-naive and castration-resistant tumors and was undetectable in lymph node metastases. These results demonstrate that *UGT2B15* and *UGT2B17* are differentially regulated during prostate cancer progression, with *UGT2B15* downregulated and *UGT2B17* upregulated, suggesting distinct roles in disease advancement and metastasis [163].

The studies involving exclusive in vitro elucidation of UGT expression and function with oncology context have employed hepatic, prostate and melanoma cancer cell lines [19,164,165,166]. Dluzen et al. established that miR216b5p represses *UGT2B7, 2B4*, and *2B10* expression in HuH7 and Hep3B liver cancer cell lines, resulting in significant decreases in glucuronidation activity against specific substrates (e.g., epirubicin, codeine, nicotine) [166]. Hu et al. (2014) characterized the regulation of *UGT2B7*, the primary epirubicin (EPI) inactivating enzyme, in response to EPI exposure [164]. Utilizing promoter construct analysis and p53 manipulation in HepG2 and Huh7 cell lines, the study inferred that EPI promotes its own detoxification by upregulating *UGT2B7* through a p53-mediated pathway involving a specific p53 response element (p53RE). This mechanism may contribute to tumor resistance and reduced cardiotoxicity compared to other anthracyclines [164]. Functional testing in LNCaP prostate cancer cells showed that miR376c negatively regulates both *UGT2B15* and 2B17 by binding to their 3′UTRs. This reduces *UGT2B15/UGT2B17* mRNA/protein levels and inhibits testosterone/androsterone glucuronidation, representing the first evidence of miRNA-mediated control for these UGTs in prostate cancer [165].

Dellinger et al. (2012) elucidated UGT expression loss and re-expression in melanoma [19]. Using primary melanoma lines (WM115, WM3211) and metastatic melanoma cell lines, the researchers inferred that while *UGT2B7*, 2B10, and 2B15 expression is lost during progression, it is inducible by anticancer agents. Crucially, *UGT2B7* knockdown sensitized WM115 cells to adriamycin/epirubicin, suggesting that inducible UGT re-expression constitutes an unsuspected mechanism for intratumoral drug resistance in melanoma [19]. Another study investigating the regulatory role of epidermal growth factor (EGF) on detoxification enzymes *UGT2B15* and *UGT2B17* in prostate cancer cells (LNCaP) found that EGF treatment suppressed the expression of *UGT2B15*, *UGT2B17*, and DNA methyltransferases DNMT3A and DNMT3B, while the inhibition of EGFR with PD16893 reversed these effects [167]. Treatment with the methyltransferase inhibitor 5-azacytidine or DNMT3B siRNA markedly reduced *UGT2B15* and *UGT2B17* expression, indicating epigenetic regulation via DNMT3B-dependent methylation. Analysis of prostate cancer versus benign prostate tissue using Illumina 450K Methylation Array data revealed differential hypomethylation of *UGT2B15* and *UGT2B17* in tumors. Collectively, the findings suggest that epigenetic dysregulation and hypomethylation of these genes may contribute to prostate cancer risk and progression, highlighting their potential as biomarkers or therapeutic targets [167].

*UGT2B7* showed two mutually exclusive exon 1 variants, each regulated by a distinct 5′ promoter. The developmental switch toward the expression of the functional enzyme was shown to occur during kidney maturation. In contrast, neoplastic cells exhibited a reversal to the inactive form, thereby showing reduced glucuronidation capacity [168]. Similarly, quantitative profiling of human renal UDP-glucuronosyltransferases revealed that neoplastic kidney tissues have markedly reduced glucuronidation capacity compared with normal kidneys, accompanied by substantial decreases in *UGT1A9* and *UGT2B7* mRNA and protein expression [169]. These findings are corroborated by Matsumoto et al., who investigated the expression and genetic variants of *UGT1A6, UGT1A9*, and *UGT2B7* in renal cell carcinoma (RCC) tissues from Japanese patients. All three UGT enzymes were significantly downregulated in RCC tissues compared with normal kidney tissue [170]. Importantly, the *UGT2B7*c.-161C>T variant and higher *UGT2B7* mRNA expression were associated with better cancer-specific survival and overall survival, respectively [170]. Thus, *UGT2B7* expression and its genetic variant were identified as independent prognostic factors, suggesting that *UGT2B7* may play a protective role in RCC progression and could serve as a prognostic biomarker.

Increased tumor expression of *UGT2B28* was associated with aggressive prostate cancer features, including higher Gleason scores, nodal invasion, and increased risk of disease progression [171]. Overexpression of the enzyme correlated with elevated circulating testosterone and dihydrotestosterone levels, whereas patients lacking *UGT2B28* gene copies showed reduced androgen and androgen–glucuronide levels with increased androstenedione [171]. These findings suggest that *UGT2B28* plays an important role in steroid metabolism, hormone homeostasis, and prostate cancer progression.

## 7. Non-Canonical Functions Independent of Metabolic Roles

The upregulation of *UGT2B17* in therapy-resistant prostate cancer has paved the way for exploring non-canonical functions. *UGT2B17* interacts with proteins involved in the unfolded protein response (UPR), thereby allowing PCa cells to withstand endoplasmic reticulum (ER) stress [172]. In addition, *UGT2B17* influences transcriptional programs linked to mitosis and the DNA damage response (DDR), mainly via Src kinase signaling. Through this pathway, it suppresses the activity of ataxia telangiectasia and Rad3-related protein (ATR) and ataxia-telangiectasia-mutated (ATM) kinases, allowing cells to bypass the G2/M checkpoint and thereby accelerating cell proliferation [172]. These observations indicate that although androgen signaling inhibition upregulates *UGT2B17* and reduces androgen dependence in PCa cells, *UGT2B17* also performs androgen-independent metabolic roles that enhance tumor cell survival and growth, ultimately contributing to the progression of castration-resistant prostate cancer (CRPC).

In Laron syndrome, where IGF1 signaling is reduced and cancer incidence is low, *UGT2B15* is markedly upregulated, suggesting that it may participate in pathways of reduced cellular stress and enhanced clearance capacity. In the same context, *UGT2B15* has been linked to p53-associated cellular defense pathways, with higher expression observed in p53 wild-type [173]. Similarly, high expression of *UGT2B17* in chronic lymphocytic leukemia is linked with poor survival and reduced drug response [174]. *UGT2B17* is shown to interact with multiple kinases within the BCR signaling pathway, including ZAP70, SYK, and BTK, suggesting a potential therapeutic vulnerability. Notably, the dual SYK and JAK/STAT6 inhibitor cerdulatinib is more effective than the selective BTK inhibitor ibrutinib in suppressing the proliferation driven by *UGT2B17* [174]. There is evidence for alternate splicing isoforms of *UGT2B7* involvement in cellular processes. Functional studies on the *UGT2B7*_i8 variant showed that alternative isoforms extend beyond traditional roles, influencing global cell metabolism [175]. The *UGT2B7*_i8 isoforms play a role within a core metabolic network, suggesting a connection between the glucuronidation pathway and primary cellular metabolic processes such as amino acid and nucleotide metabolism [175]. It remains to be clarified whether this link involves protein interactions (e.g., dimerization/oligomerization) or the glucuronidation of novel endogenous substrates. Additionally, *UGT2B7*_i8 expression has also been shown to influence cell adhesion and proliferation, reinforcing the idea that UGT enzymes play broader roles in regulating multiple cellular functions beyond detoxification [175].

## 8. Future Directions

*UGT2B* family members play a significant role in the metabolism of endogenous substrates, and drugs, whose function in turn is affected by drugs and endogenous substances. Future research must focus on the tissue-specific distribution and non-canonical roles of these highly expressed enzymes in addition to their metabolic role. Furthermore, exploiting drug–drug interactions mediated by *UGT2B* members to improve the efficacy of the substrates must be undertaken. The compensatory role of other *UGT2B* members in the absence of closest members and dual protein–protein interactions of *UGT2B* members may also shed light on how we can exploit their metabolic role and tissue-specific interactions for improved efficacy and reduced toxicities in oncology setting.

*UGT2B17* has emerged as a potential therapeutic target in pediatric cancer treatment due to its involvement in oncogenic signaling pathways and drug resistance. *UGT2B17* has a non-canonical role as part of the BCR signalosome, interacting with kinases such as ZAP70, SYK, and BTK. These interactions enhance prosurvival and proliferative signaling in leukemic cells. Dual inhibition of SYK and JAK/STAT6 pathways using agents like cerdulatinib has shown promise in overcoming the proproliferative effects of *UGT2B17*. This approach is more effective compared to selective BTK inhibitors like ibrutinib, which fail to fully abrogate *UGT2B17*-mediated proliferation [174]. *UGT2B17* is also connected to IL4 cytokine signaling, which contributes to its oncogenic functions. Targeting the JAK/STAT6 pathway could disrupt this signaling axis, reducing the survival advantage conferred by *UGT2B17* overexpression [155].

High expression of *UGT2B17* correlates with poor prognosis and resistance to chemotherapy in certain cancers like chronic lymphocytic leukemia (CLL). Epigenetic therapies or gene editing approaches could be employed to downregulate *UGT2B17* expression or disrupt its transcriptional regulation, potentially improving treatment outcomes [155]. Since *UGT2B17* enhances resistance to chemotherapeutic agents, combining standard chemotherapy with inhibitors targeting *UGT2B17*-mediated pathways (e.g., SYK/JAK or BCR signaling) may improve efficacy. For example, combining cerdulatinib with conventional therapies could sensitize resistant cancer cells to treatment [176].

The expression level of *UGT2B17* can serve as a biomarker for identifying patients at higher risk of poor outcomes or those less likely to respond to specific treatments. Stratifying patients based on *UGT2B17* expression could guide personalized treatment plans, optimizing therapeutic choices.

In summary, therapeutic strategies targeting *UGT2B17* focus on disrupting its role in oncogenic signaling pathways (e.g., BCR and JAK/STAT6), modulating its expression, and integrating it into combination therapies. These approaches hold the potential for improving outcomes in pediatric cancers where *UGT2B17* plays a critical role.

## 9. Conclusions

The evidence discussed in this review reinforces the importance of the *UGT2B* enzyme family in cancer biology and treatment, particularly in hormone-driven malignancies and cancers in which drug metabolism strongly influences therapeutic response. Across different cancer types, genetic variation, tissue-specific expression, and complex regulatory mechanisms, including epigenetic and microRNA-mediated control, shape both disease risk and treatment outcomes. While *UGT2B* enzymes are best known for their role in the glucuronidation of endogenous compounds and anticancer drugs, accumulating data indicate that certain isoforms, such as *UGT2B17*, may also contribute to oncogenic signaling and treatment resistance in a context-dependent manner. This dual metabolic and functional relevance has clear translational implications. From a therapeutic perspective, targeting *UGT2B* activity should not be viewed in isolation, but rather as part of rational combination strategies. Selective inhibition or modulation of *UGT2B* family members may enhance the effectiveness of co-administered anticancer agents without increasing their dose, while simultaneously allowing the dose reduction in drugs associated with dose-dependent toxicities. Taken together, these observations place *UGT2B* enzymes as clinically relevant modifiers of cancer treatment and support their further evaluation as biomarkers and therapeutic targets within precision oncology frameworks.

## Data Availability

No new data were created or analyzed in this study. Data sharing is not applicable to this article.

## Supplementary Materials

The following supporting information can be downloaded at https://www.mdpi.com/article/10.3390/ph19071016/s1; Figure S1: *UGT2B* family members’ genetic variations across various cancers. A minimum of 100 patients were considered as an inclusion criterion for obtaining frequency data. Various cohorts and their original sources of the data can be seen on the plots, and can also be obtained from the cBioPortal webpage. (A) UGT2B17 variations across cancer types; (B) UGT2B15 variations across cancer types (minimum 100 patients per cancer type); (C) UGT2B7 variations across cancer types (minimum 100 patients per cancer type); (D) UGT2B10 variations across cancer types (minimum 100 patients per cancer type) [177]; Table S1: Cooccurrence tendencies of variants in *UGT2B* family members across the datasets investigated in cBioPortal; Table S2: A comparison of allele frequencies in *UGT2B* family genes in various populations. The data presented are not comprehensive and are only meant to indicate the population variations seen [104,178,179,180,181,182,183,184,185,186,187,188].
