# Supplementary material for: Roles of Uridine Diphosphoglucuronosyltransferase 2B Enzymes in Cancer Susceptibility and Treatment: A Review
Source: Pharmaceuticals (Basel). 2026 Jun 30;19(7):1016. doi: 10.3390/ph19071016 (PMC13416403; doi:10.3390/ph19071016)
Supplement: Supplementary file 1 [file pharmaceuticals-19-01016-s001.zip › pharmaceuticals-4284005-supplementary.pdf]

pharmaceuticals

Review

Roles of Uridine diphosphoglucuronosyltransferase 2B Enzymes in Cancer Susceptibility and Treatment: A Review

Suresh Kumar Srinivasamurthy[1], Vijaya Paul Samuel[2], Tarig Hakim Merghani Hakim[3], Biji Thomas George[4], Grisilda Vidya Bernardt[5], Ashwin Kamath[6], Uppugunduri Satyanarayana Chakradhara Rao[7,*]

[1] Department of Pharmacology, RAK College of Medicine, RAK Medical and Health Sciences University, Ras Al Khaimah, UAE suresh@rakmhsu.ac.ae
[2] Department of Anatomy, RAK College of Medicine, RAK Medical and Health Sciences University, Ras Al Khaimah, UAE vijaypaul@rakmhsu.ac.ae
[3] Department of Physiology, RAK College of Medicine, RAK Medical and Health Sciences University, Ras Al Khaimah, UAE tarig@rakmhsu.ac.ae
[4] Department of Surgery, RAK College of Medicine, RAK Medical and Health Sciences University, Ras Al Khaimah, UAE biji@rakmhsu.ac.ae
[5] Department of Biochemistry, RAK College of Medicine, RAK Medical and Health Sciences University, Ras Al Khaimah, UAE grisilda@rakmhsu.ac.ae
[6] Department of Pharmacology, Kasturba Medical College Mangalore, Manipal Academy of Higher Education, Manipal, India ashwin.kamath@manipal.edu
[7] Department of Medical Oncology, Jawaharlal Institute of Postgraduate Medical Education and Research, Puducherry, India

* Correspondence: uscrao@jipmer.ac.in ; Tel.: (optional; include country code; if there are multiple corresponding authors, add author initials)

Figure S1: UGT2B family members genetic variations across various cancers. A minimum of 100 patients were considered as an inclusion criterion for obtaining frequency data. Various cohorts and their original sources of the data can be seen on the plots, and also can be obtained from CBioportal webpage [177].

A) UGT2B17 variations across cancer types.

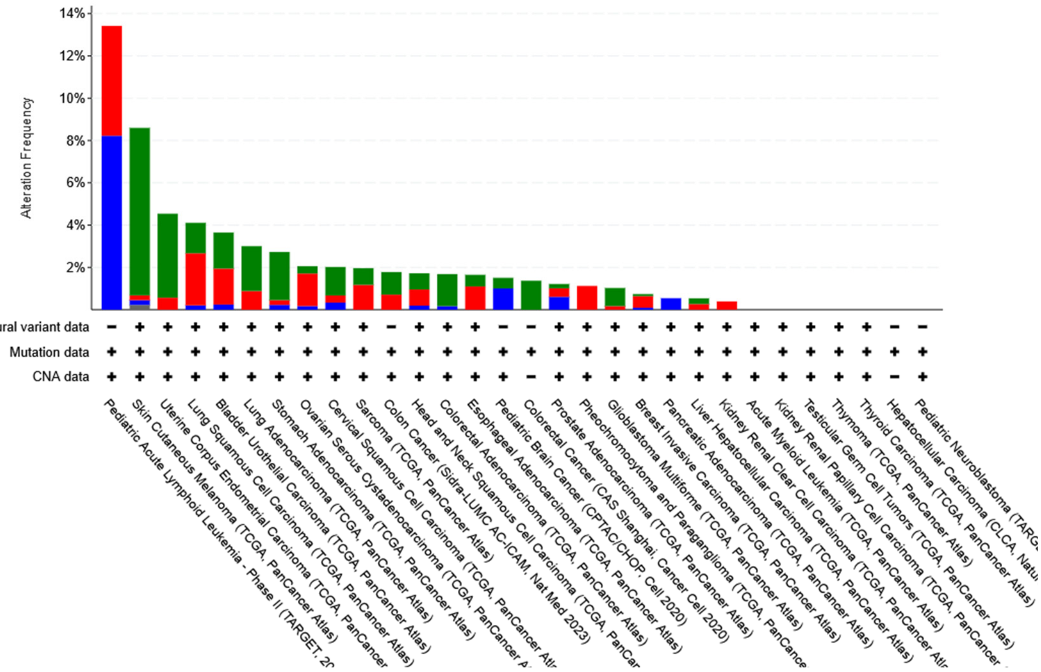

B) UGT2B15 variations across cancer types (minimum 100 patients per cancer type)

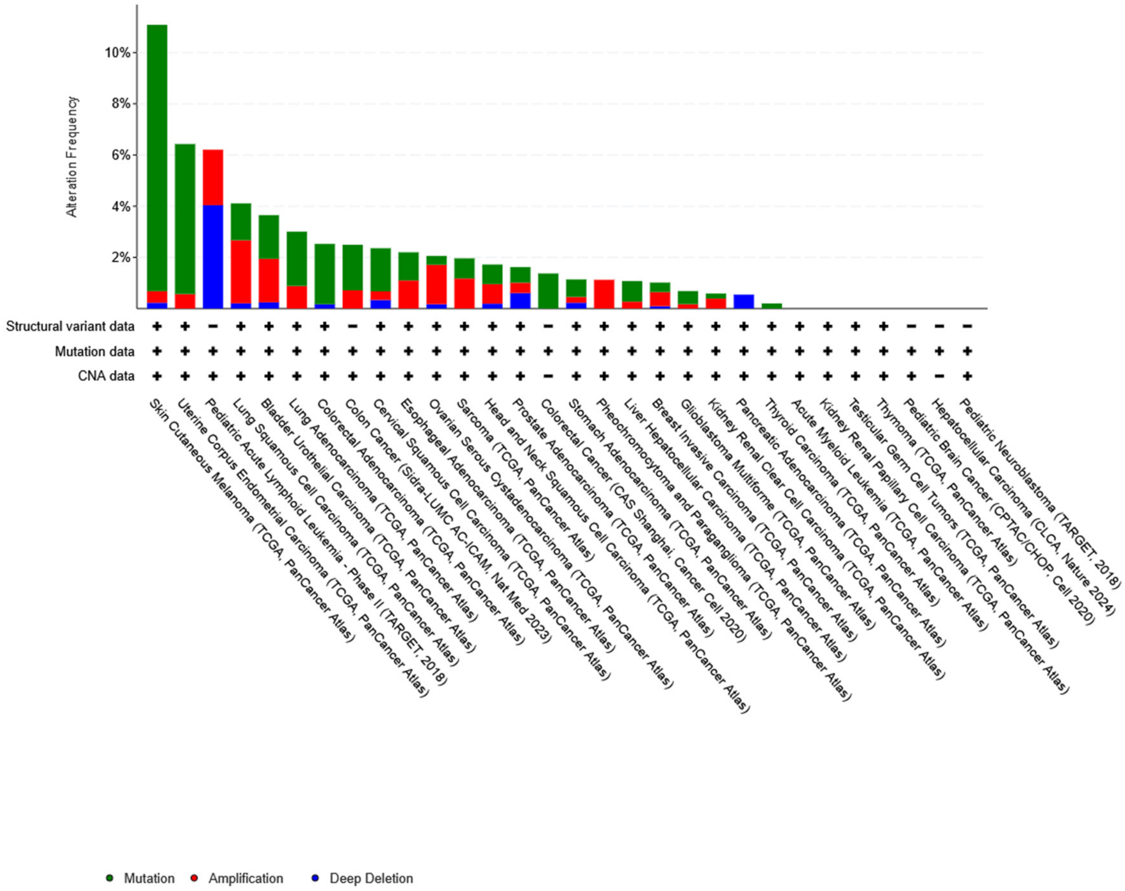

C) UGT2B7 variations across cancer types (minimum 100 patients per cancer type)

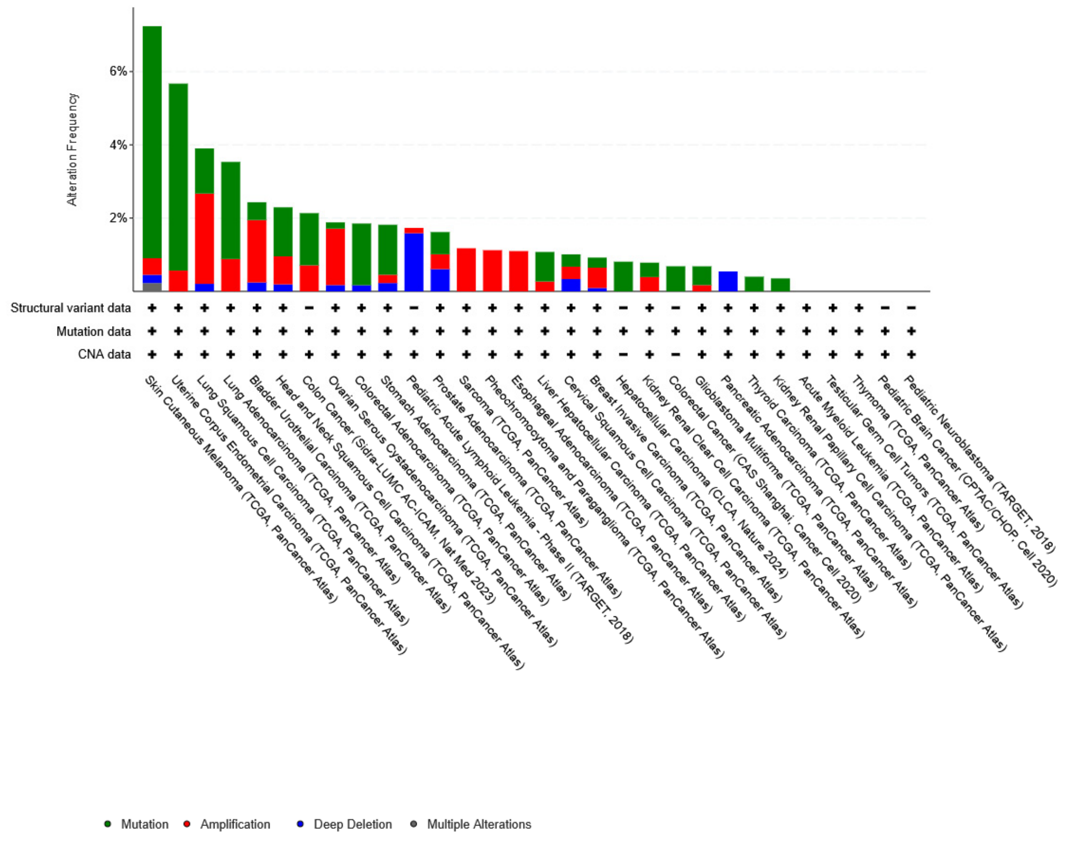

D) UGT2B10 variations across cancer types (minimum 100 patients per cancer type)

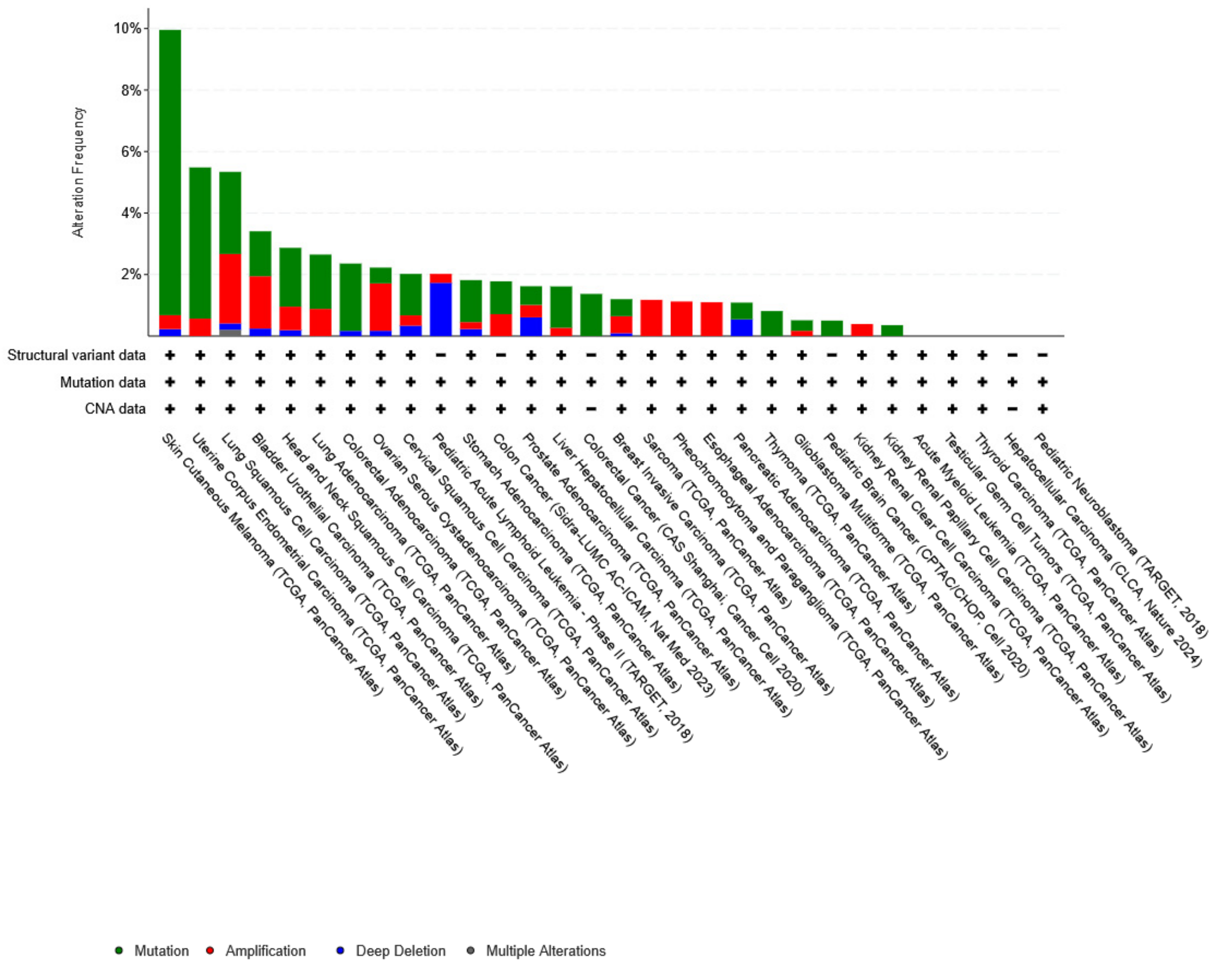

Table S1: Cooccurrence tendencies of variants in UGT2B family members across the datasets investigated in Cbioportal.

**A**	**B**	**Neither**	**A Not B**	**B Not A**	**Both**	**Log2 Odds Ratio**	**pValue**	**qValue**	**Tendency**
UGT2B10	UGT2B7	11484	129	102	108	>3	<0.001	<0.001	Cooccurrence
UGT2B15	UGT2B17	11476	130	112	105	>3	<0.001	<0.001	Cooccurrence
UGT2B7	UGT2B15	11478	110	135	100	>3	<0.001	<0.001	Cooccurrence
UGT2B10	UGT2B15	11453	135	133	102	>3	<0.001	<0.001	Cooccurrence
UGT2B10	UGT2B17	11465	141	121	96	>3	<0.001	<0.001	Cooccurrence
UGT2B7	UGT2B17	11487	119	126	91	>3	<0.001	<0.001	Cooccurrence

Table S2. A comparison of allele frequencies in UGT2B family genes in various populations. The data presented are not comprehensive and are only meant to indicate the population variations seen.

**Study**	***Gene***	**Variants studied**	**Population**	**Minor allele frequency (%)**
Ford et al. 2022 [178]	*UGT2B7*	c.802C>T (rs7439366)	African	77.49
			American	68.01
			European	51.49
			East Asians	72.52
			South Asians	60.12
Božina et al. 2023 [179]	*UGT2B7*	c.-161C>T (rs7668258)	Croatians	50.60
			African/African American	29.22
			Latino/Admixed American	31.04
			Finnish	44.01
			Non-Finnish Europeans	53.76
			East Asians	29.43
			South Asians	39.90
			Ashkenazi Jewish	43.75
Bertholim-Nasciben et al. 2023 [180]	*UGT2B17*	UGT2B17 NULL allele	Brazilian	34.30
Hwang et al. 2014 [181]	*UGT2B15*	c.-1139T>C (rs9994887)	Korean	42
		c.-508G>A (rs1120265)	Korean	42
		c.-506T>A (rs1580083)	Korean	42
		c.253T>G (rs1902023)	Korean	42
		c.23687A>T (rs4148271)	Korean	31
		c.2635A>T (rs2045100)	Korean	28
		c.-497C>T OR 378 C>T (rs4148269)	Korean	14
		c.23669C>T (rs1902023)	Korean	12
		c.23476A>C (rs4148269)	Korean	11
Mehlotra et al. 2006 [182]	*UGT2B7*	c.211G>T	Caucasian-American	0
			African-American	0
			Asian-American	9
			Hispanic-American	2
			West African	0
			Papua New Guinean	0
		c.802C>T (rs7439366)	Caucasian-American	52
			African-American	34
			Asian-American	41
			Hispanic-American	28
			West African	21
			Papua New Guinean	28

Kim et al. 2014 [183]	*UGT2B7*	c.802C>T (rs7439366)	Korean	32.3
			Han Chinese	25.5
			Japanese	27.1
			European American	46.8
Roco et al. 2012 [184]	*UGT2B7*	c.802C>T (rs7439366)	Chilean	40
			Caucasian	49
			Japanese	27
Park et al. [104]	*UGT2B17*	Deletion polymorphism	Caucasian	11
			African American	12
John et al. 2024 [185]	*UGT2B15*	c.253 G>T (rs1902023)	Thai	53
			East Asian	55
			African	59
			Admixed American	61
			Middle East	50
			European	47
			South Asian	44
		c.-497 C>T (rs4148269)	Thai	28
			East Asian	17
			African	20
			Admixed American	53
			Middle East	54
			European	65
			South Asian	52
Wilson et al. 2004 [186]	*UGT2B17*	Deletion	Caucasian	11
			African American	2
Hwang et al. 2010 [187]	*UGT2B7*	*2 (rs7439366)	Korean	39
			Japanese	25
			Chinese	33
			Asian	27
			Caucasian	49
			Norwegian	56
Bhasker et al. 2000 [188]	*UGT2B7*	*2 (rs7439366)	Caucasian	48.9
			Japanese	26.8
